# Molecular dynamics at immune synapse lipid rafts influence the cytolytic behavior of CAR T cells

**DOI:** 10.1126/sciadv.adq8114

**Published:** 2025-01-10

**Authors:** Ahmed Z. Gad, Jessica S. Morris, Lea Godret-Miertschin, Melisa J. Montalvo, Sybrina S. Kerr, Harrison Berger, Jessica C. H. Lee, Amr M. Saadeldin, Mohammad H. Abu-Arja, Shuo Xu, Spyridoula Vasileiou, Rebecca M. Brock, Kristen Fousek, Mohamed F. Sheha, Madhuwanti Srinivasan, Yongshuai Li, Arash Saeedi, Kandice R. Levental, Ann M. Leen, Maksim Mamonkin, Alexandre Carisey, Navin Varadarajan, Meenakshi Hegde, Sujith K. Joseph, Ilya Levental, Malini Mukherjee, Nabil Ahmed

**Affiliations:** ^1^ Interdepartmental Translational Biology and Molecular Medicine Graduate Program, Baylor College of Medicine, Houston, TX 77030, USA.; ^2^ Texas Children’s Cancer Center, Texas Children’s Hospital, Baylor College of Medicine, Houston, TX 77030, USA.; ^3^ Center for Cell and Gene Therapy, Texas Children’s Hospital, Houston Methodist Hospital, Baylor College of Medicine, Houston, TX 77030, USA.; ^4^ Department of Pediatrics, Baylor College of Medicine, Houston, TX 77030, USA.; ^5^ Dan L. Duncan Comprehensive Cancer Center, Baylor College of Medicine, Houston, TX 77030, USA.; ^6^ William A. Brookshire Department of Chemical and Biomolecular Engineering, University of Houston, Houston, TX 77204, USA.; ^7^ Immunology & Microbiology Graduate Program, Baylor College of Medicine, Houston, TX 77030, USA.; ^8^ Texas Children’s Hospital William T. Shearer Center for Human Immunobiology, Houston, TX 77030, USA.; ^9^ Development, Disease Models & Therapeutics Graduate Program, Baylor College of Medicine, Houston, TX 77030, USA.; ^10^ Department of Molecular Physiology and Biological Physics, Center for Molecular and Cell Physiology, University of Virginia, Charlottesville, VA 22903, USA.; ^11^ Department of Medicine, Baylor College of Medicine, Houston, TX 77030, USA.; ^12^ Department of Pathology and Immunology, Baylor College of Medicine, Houston, TX 77030, USA.

## Abstract

Chimeric antigen receptor T cells (CART) targeting CD19 through CD28.ζ signaling induce rapid lysis of leukemic blasts, contrasting with persistent tumor control exhibited by 4-1BB.ζ-CART. We reasoned that molecular dynamics at the CART immune synapse (CARIS) could explain differences in their tumor rejection kinetics. We observed that CD28.ζ-CART engaged in brief highly lethal CARIS and mastered serial killing, whereas 4-1BB.ζ-CART formed lengthy CARIS and relied on robust expansion and cooperative killing. We analyzed CARIS membrane lipid rafts (mLRs) and found that, upon tumor engagement, CD28.ζ-CAR molecules rapidly but transiently translocated into mLRs, mobilizing the microtubular organizing center and lytic granules to the CARIS. This enabled fast CART recovery and sensitivity to low target site density. In contrast, gradual accumulation of 4-1BB.ζ-CAR and LFA-1 molecules at mLRs built mechanically tonic CARIS mediating chronic Fas ligand–based killing. The differences in CD28.ζ- and 4-1BB.ζ-CARIS dynamics explain the distinct cytolytic behavior of CART and can guide engineering of more adaptive effective cellular products.

## Teaser

CAR signaling domains determine molecular dynamics at the immune synapse and consequently influence CAR T cell killing behavior.

## INTRODUCTION

Chimeric antigen receptor (CAR) T cells are currently US Food and Drug Administration (FDA) approved for B cell leukemia, lymphoma, and multiple myeloma (*1*). In contrast, although several promising CAR T cells that target solid tumors are in the pipeline, sustained clinical responses have been limited (*2*, *3*). Greater understanding of CAR T cell function at the cellular and molecular levels could help extend our success beyond lymphoreticular malignancies.

Despite the efficacy of CD28- and 4-1BB–signaling CAR (CAR^CD28ζ^ and CAR^4-1BBζ^, respectively) T cells targeting CD19 on B cell malignancies in the clinic, their patterns of efficacy and toxicity are distinct (*4*). In the short term, CD19-CAR^CD28ζ^ T cells exhibit more robust activation and more severe cytokine-mediated complications (*5*–*8*), whereas in the long term, the progression-free survival is more sustained with CD19-CAR^4-1BBζ^ T cells in B cell leukemia due to steadier antitumor activity and T cell longevity (*9*–*15*). Nonetheless, CD19-CAR^CD28ζ^ T cells are more clinically effective against various types of B cell lymphoma, where intense initial cytolysis seems to be very important (*16*–*18*).

In solid cancers, clinical data are inadequate for meaningful comparisons of CAR designs, but animal studies suggest that CAR^CD28ζ^ T cells targeting GPC-2, mesothelin, IL13Rα2, B7-H3, and HER2 produce more rapid control of neuroblastoma, ovarian cancer, glioma, osteosarcoma/glioma, and osteosarcoma, respectively (*19*–*24*). Most of these responses were more prone to relapses than those treated with CAR^4-1BBζ^ T cells (*19*–*24*). These preclinical data, together with clinical findings from CD19-targeting studies in B-lineage leukemia and lymphoma, highlight the role of tumor rejection kinetics mediated by different signaling domains and their importance in achieving durable tumor control.

Cellular kinetic studies, of both target and effector cells, have shown that several covariates contribute to the efficacy of CAR T cells, independent of the infused doses (*25*–*31*). At the cellular level, CAR binding affinity and target density were explored, and modulated, as determinants of CAR T cell efficacy (*23*, *32*–*37*). However, CAR T cells express a plethora of molecules that interact with cognate receptors on tumor cells at their interface, known as the CAR immune synapse (IS), and influence their structural avidity (*38*–*43*). This structural avidity is distinct from the manner in which the CAR IS (CARIS) influences the CAR T cell activation and cytotoxic response, which is referred to as their functional avidity (*43*, *44*).

The biology of synapse formation by the T cell receptor (TCR) has been studied extensively (*45*, *46*). The role of CARIS in determining CAR T cell efficacy is ill defined. CAR T cells form nonclassical IS in which CAR molecules, costimulatory, co-inhibitory, and adhesion molecules are not distributed in the concentric supramolecular activation clusters as in TCR IS (*47*–*49*). Similar to TCR IS, however, CARIS formation entails polymerization of actin microfilaments, polarization of the microtubule organizing center (MTOC), and convergence of lytic granules, mediating target cell killing (*47*, *50*). These molecular dynamics could be influenced by the differences between CD28 and 4-1BB at the CARIS level (*49*, *51*).

An association between the TCR IS and the cholesterol- and sphingolipid-rich membrane microdomains termed membrane lipid rafts (mLRs) has been established and substantiated (*52*–*63*). Specifically, mLRs have been shown to facilitate the recruitment of the TCR and costimulatory, coinhibitory, and cell adhesion molecules to the TCR IS (*52*–*63*). This important association remains to be established in CAR T cells because it represents an opportunity to study the molecular events and dynamics thereof at the CARIS and how these relate to the structural and functional avidity of CAR T cells.

Here, we attempt to determine whether events taking place at the CARIS mLRs could explain the cytolytic behavior of CAR T cells upon encountering cancer cells. A better understanding of both the structural and functional dynamics at the CARIS can help in designing CAR T cell therapies with improved reactivity to resistant tumors and possibly reduced toxicity.

## RESULTS

### CAR^CD28ζ^ T cells engage in brief acutely lethal CARIS allowing for increased serial killing

**Fig. 1.**
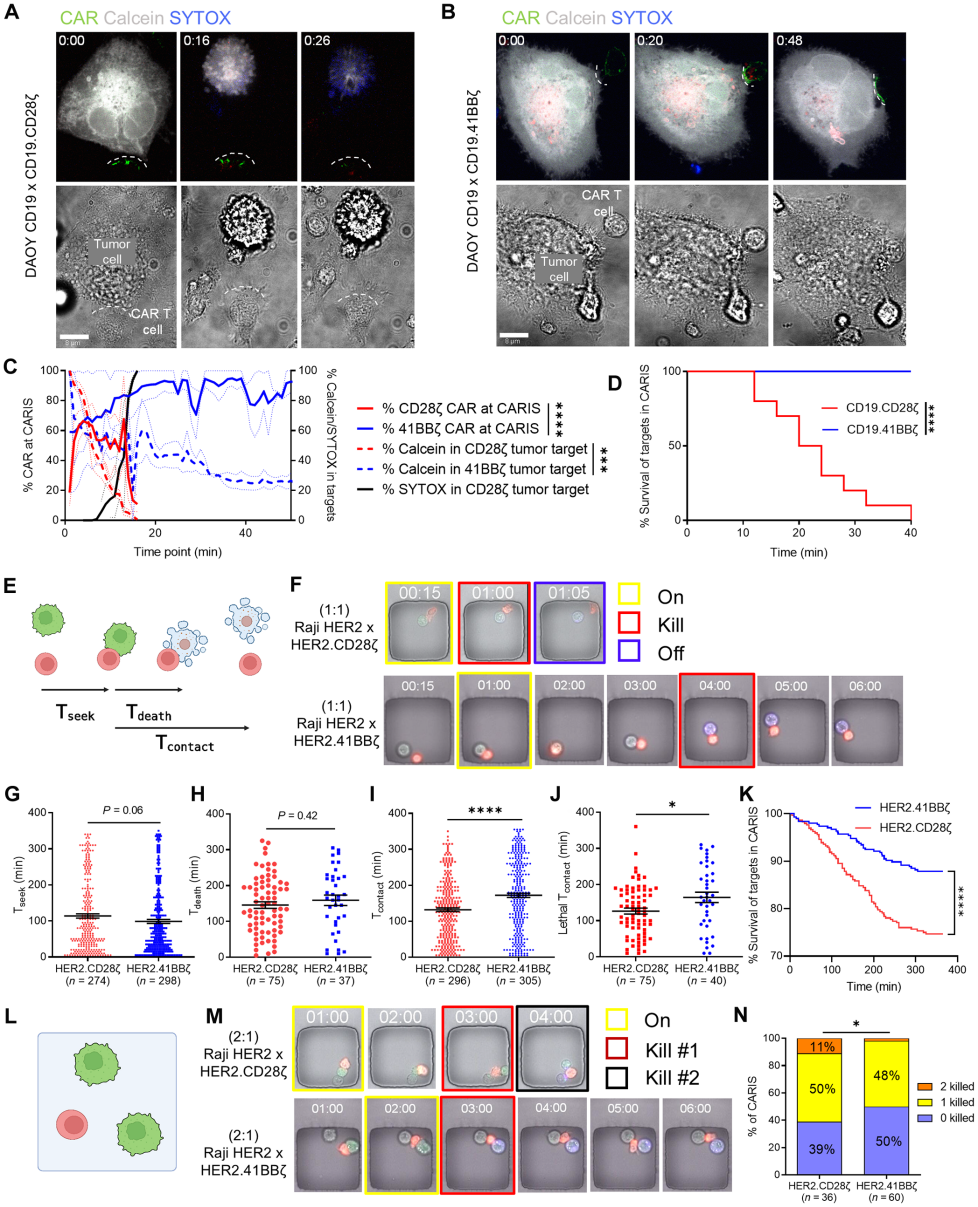
Dynamics of individual CAR T cell killing of target cells. (**A** and **B**) Time-lapse microscopy of CD19-CAR^CD28ζ^ (A) and CD19-CAR^4-1BBζ^ (B) T cells conjugated with Daoy.CD19 target cells. Time zero represents formation of a CAR and target cell conjugate. Green, emerald GFP CAR; blue, SYTOX viability dye (uptake indicates target cell death); silver, calcein AM viability dye (loss indicates target cell death). (**C**) Quantification of percent CAR at the CARIS in CD19-CAR^CD28ζ^ (red solid line) and CD19-CAR^4-1BBζ^ (blue solid line) T cells and the corresponding %Calcein (red dashed line) and %SYTOX (black solid line) in target cells conjugated to CD19-CAR^CD28ζ^ and %Calcein in target cells conjugated to CD19-CAR^4-1BBζ^ T cells (blue dashed line); mean + SEM. [(A) to (C)], *n* = 5. (**D**) Percentage survival for Daoy.CD19 conjugated to CD19-CAR^CD28ζ^ or CD19-CAR^4-1BBζ^ T cells. *n* = 20. (**E**) Schematic representation for TIMING assay. HER2-CAR T cells (PKH26 Red cell membrane label); Raji.HER2 tumor cells (GFP tagged/PKH67 Green cell membrane label) at 1:1. Annexin V (blue) marker for tumor cell death. (**F**) Filmstrip for representative images of the TIMING assay at 1:1. Yellow box, initial contact; red box, target death; blue box, detachment. (**G**) T_seek;_ (**H**) T_death;_ and (**I**) T_contact_ of all encounters. (**J**) T_contact_ of cytolytic encounters. (**K**) Percentage of Raji.HER2 survival after contact with HER2-CAR T cells. *n* = 600. (**L**) Schematic of serial killing experiment. (**M**) Filmstrip for representative images of HER2-CAR T cells and Raji.HER2 at 1:2. Yellow box initial contact; red box, target cell death; black box, death of both target cells. (**N**) Percentage of CARIS that lead to 0, 1, or 2 target cell deaths at 1:2. One-way ANOVA of areas under the curve with Holm-Sidak corrected multiple comparisons (C), log-rank (Mantel-Cox) test [(D) and (K)], Student’s *t* test [(G) to (J)], or χ^2^ test (N). Representative of two to three donors [(A) to (D)] or data from two donors pooled [(F) to (N)]. *P < 0.05, ***P < 0.001,****P < 0.0001. (E) and (L) were created using Biorender.com.

We studied the interaction dynamics and lethality of individual CAR^CD28ζ^ and CAR^4-1BBζ^ T cells against CD19- and HER2-expressing target cells using time-lapse live microscopy (Fig. 1 and fig. S1, A to D). We simultaneously quantified CAR aggregates at the CARIS and target cell death over time and observed rapid accumulation followed by rapid reduction in CD19-CAR^CD28ζ^ clusters at the CARIS. Within 20 min, we observed complete loss of calcein and a SYTOX signal in target cells, indicating target cell death (Fig. 1A and movie S1). In contrast, we observed a continued increase in CD19-CAR^4-1BBζ^ aggregation at the CARIS past 50 min associated with a slow and incomplete loss of calcein over this time period (Fig. 1B and movie S2; both Figs. 1A and 1B are quantified in Fig. 1C). We observed a significant difference in killing of target cells, with near-complete elimination of targets by CD19-CAR^CD28ζ^ T cells and limited killing by the CD19-CAR^4-1BBζ^ T cells within 40 min of testing (Fig. 1D).

To validate these single-cell findings in high-throughput assays over a longer time period, we used time-lapse imaging microscopy in nanowell grids (TIMING; Fig. 1E) (*64*), which can assess some characteristics and quantify single-cell interactions. We used HER2-CAR T cells against Raji (lymphoma cell line) that was engineered to express HER2 to test parameters that necessitate the target to be mobile. Namely, we compared the time needed by CAR T cells to seek target cells (T^seek^), the duration in contact (T^contact^), and the time to achieve target death (T^death^) once effector-target conjugation is initiated. At a 1:1 effector-to-target ratio, HER2-CAR^CD28ζ^ and HER2-CAR^4-1BBζ^ T cells had comparable T^seek^ and T^death^, indicating their similar dynamics in achieving target killing (Fig. 1, F to H, and movie S3). HER2-CAR^CD28ζ^ T cells had significantly shorter T^contact^ in all synapse events (Fig. 1I) and in lethal encounters (Fig. 1J). Over 6 hours, HER2-CAR^CD28ζ^ T cells achieved significantly more lethal cytolytic CARIS than HER2-CAR^4-1BBζ^ T cells (Fig. 1K). Last, we observed that, when single HER2-CAR^CD28ζ^ T cells encountered more than one target (Fig. 1L), they exhibited a significantly higher frequency of second or subsequent cytolytic CARIS, supporting their ability to “serially kill” targets (Fig. 1, M and N, and movie S4). We observed that a single HER2-CAR^CD28ζ^ T cell can engage in two cytolytic CARIS simultaneously and had a higher overall total number of targets killed per effector.

We therefore concluded that the superior short-term killing capacity of CAR^CD28ζ^ T cell products could be attributed to the faster-paced engagement dynamics and higher “serial killing” capacity of individual CAR^CD28ζ^ T cells. Although individual CAR^4-1BBζ^ T cells engaged in a lengthier killing process, their cytolytic efficacy in long-term assays was comparable to that of CAR^CD28ζ^ T cells (fig. S1, E to H), which was attributed to antigen-specific stimulation, rather than alloreactivity that could be triggered by prolonged interaction (fig. S2). In these experiments, the FDA-approved CAR designs were used to relate our findings to the clinicopathological presentation of CD19-CAR T cells (*65*).

### Sustained killing by CAR^4-1BBζ^ T cells is proportionate to their proliferation and is collaborative in nature

We reasoned that the ability of CAR^4-1BBζ^ T cells to compensate for their short-term killing disadvantage over time could be attributed to their proliferative capacity. We computed a rate constant (ρ_cytotox_) for CAR T cell fold expansion (fold_x_) in relation to their cytotoxicity using the formula

$$\rho_{\text{cytotox}}=\text{log}({\text{fold}}_{x})/{\text{Cytotox}}_{\text{max}}$$

where Cytotox_max_ is the percentage cytotoxicity at peak expansion.

**Fig. 2.**
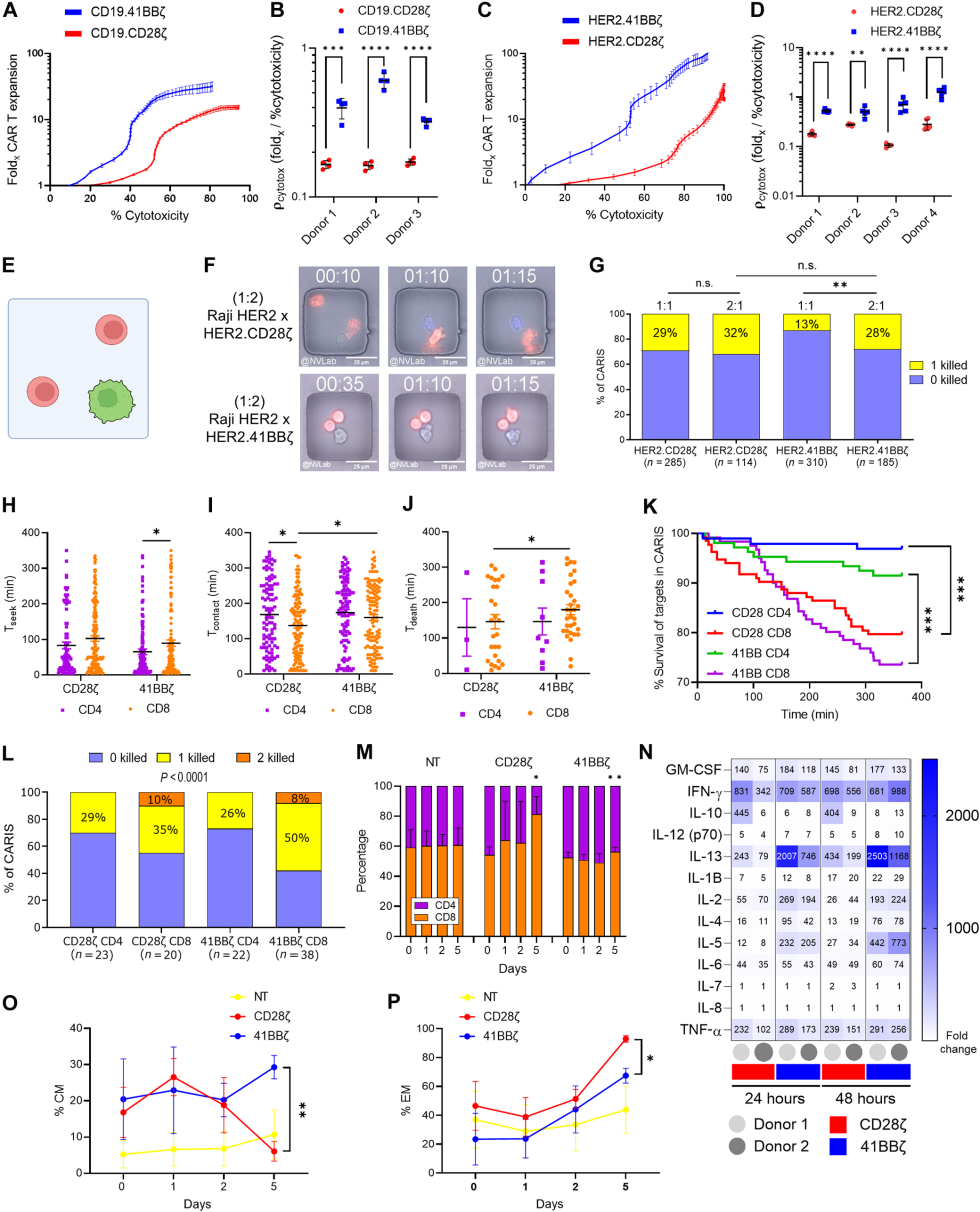
CAR T cell expansion, phenotypic shift, and relationship to kinetics of target cell killing. (**A** to **D**) T cell tracking synchronized with tumor cell tracking of CD19-CAR T cells versus Daoy.CD19 (A) and HER2-CAR T cells versus LN229 (C). Rate constant (ρ_cytotox_) for CAR T cell fold expansion (fold_x_) in relation to their cytotoxicity for CD19-CAR T cells (B) and HER2-CAR T cells (D). Dot, technical replicate. (**E** and **F**) Schematic (E) and filmstrip (F) of the TIMING cooperative/additive killing experiment of HER2-CAR T cells and Raji.HER2 at 2:1. (**G**) Percentage of CARIS that lead to 0 or 1 target cell death (two HER2-CAR T cells made contact with one Raji.HER2), compared to the killing at 1:1. (**H** to **K**) TIMING: T_seek_ (H), T_contact_ (I), and T_death_ (J) and percentage of Raji.HER2 survival after CAR T cell contact (K) in wells having CD4 versus CD8 of HER2-CAR T cells and Raji.HER2 at 1:1. *n* = 457. (**L**) Percentage of events that lead to 0, 1, or 2 target cell deaths in wells that have one CD4 or CD8 HER2-CAR T cell that contacted two tumor targets. (**M** to **P**) Flow cytometry for CD4/CD8 polarization of HER2-CAR T cells or NT (M) and fold change of cytokine secretion (MILLIPLEX) to NT done on the supernatant collected after 24 and 48 hours (N) and change in percentage of CM (O) and EM (P) for T cells (50/50 CD4:CD8) cocultured with LN229. One-way ANOVA [(B), (D), (H), (I), and (J)] or repeated measures (RM) two-way ANOVA [(M), (O), and (P)] with Holm-Sidak corrected multiple comparisons, χ^2^ test [(G) and (L)], or log-rank (Mantel-Cox) test (K). Representative of three to four donors [(A) and (C); single donor values depicted in (B) and (D), respectively], pooling of data from two donors [(E) to (L)], or the mean and SD for three donors [(M), (O), and (P)]. n.s. = not significant, *P < 0.05, **P < 0.01, ***P < 0.001, ****P < 0.0001. (E) was created using Biorender.com.

CAR^4-1BBζ^ T cells were more proliferative upon tumor encounter and had significantly higher ρ_cytotox_ when compared to CAR^CD28ζ^ T cells, reflecting a higher dependency on expansion to achieve the same cytotoxicity (Figs. 2A and 2C and Figs. 2B and 2D represent ρ_cytotox_ values computed from the curves in Fig. 2A and Fig. 2C, respectively; fig. S3, A to F).

Next, we assessed whether CAR^4-1BBζ^ T cells collaborated in killing target cells. We examined, in the TIMING assay, a scenario where effectors exist in excess (Fig. 2E) and observed that the presence of additional effector cells improved the efficiency of killing of single target cells (Fig. 2, F and G, movie S5). This indicated that HER2-CAR^4-1BBζ^ T cells had a tendency toward “cooperative killing” of individual targets. We also observed that multiple CAR^4-1BBζ^ T cells engaged a single tumor target cell simultaneously. In contrast, an excess of HER2-CAR^CD28ζ^ T cells did not improve target killing.

Because the CD4+:CD8+ composition was shown to influence the antitumor kinetics of CAR T cells (*66*–*68*), we tested the cytotoxic behavior of individual CD4 and CD8 CAR T cells in TIMING assay. We found that, in both CD28- and 4-1BB–signaling CAR T cells, not only CD8 CAR T cells had a significantly shorter T^contact^ (Fig. 2, H and I) than CD4’s but also CD8 CAR^CD28ζ^ T cells had significantly shorter T^death^ (Fig. 2J). Moreover, CD8 T cells were more cytotoxic and had better serial killing rates, with no difference between CAR^CD28ζ^ and CAR^4-1BBζ^ T cells (Fig. 2, K and L, and fig. S3, G and H). Accordingly, we reasoned that the acute killing capacity of CAR^CD28ζ^ T cells could be driven by a change in the phenotype of the T cell population to favor the growth of the more cytotoxic CD8 subset, i.e., CD8 polarization (*69*). We mixed CD4 and CD8 CAR T cells at a 1:1 ratio prior to coculture with target cells and observed a notable increase in the frequency of CD8+ of the CAR^CD28ζ^ T cells as early as 24 hours of coculture with tumor cells. In contrast, CAR^4-1BBζ^ T cells maintained a balance of CD4/CD8 T cells over time (Fig. 2M).

CAR^4-1BBζ^ T cells secreted significantly higher levels of interleukin-2 (IL-2), IL-4, IL-5, IL-6, and IL-13 (Fig. 2N and fig. S3I). These T helper 2 (T_H_2) cytokines could explain the robust expansion of CD4 T cells in HER2-CAR^4-1BBζ^ products, which was also associated with a significantly larger population of central memory (CM) phenotype, contrasting with a predominance of effector memory (EM) in HER2-CAR^CD28ζ^ T cells (Fig. 2, O and P, and fig. S3J). The analysis of CD19-CAR T cells showed comparable results (fig. S3, K to N).

In summary, the steady killing behavior of CAR^4-1BBζ^ T cells was proportionate to their expansion. They preserved CD4 effectors, a more CM phenotype, and showed evidence of cooperative killing.

### CAR shuttling to lipid rafts underpins synapse dynamics of effector T cells

**Fig. 3.**
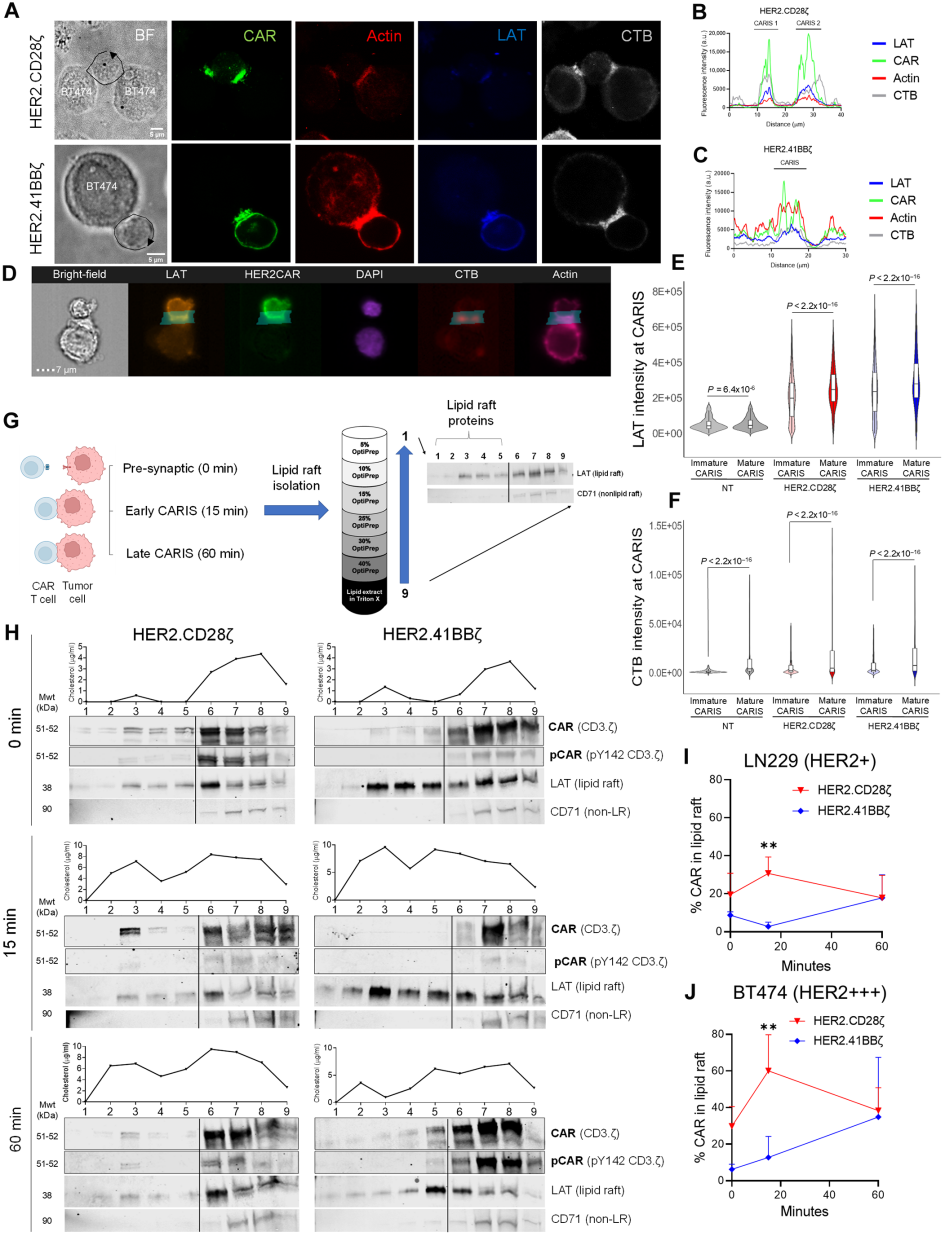
Association of CAR molecules with mLRs. (**A**) Confocal microscopy of HER2-CAR T cell conjugates with the HER2-amplified breast cancer line, BT474, imaged for CARs [emerald GFP (green)], actin [phalloidin (red)], LAT (blue), and CTB (gray). Distribution of the fluorescence intensity on the membrane of (**B**) HER2-CAR^CD28ζ^ and (**C**) HER2-CAR^4-1BBζ^ T cells conjugates in arbitrary units (a.u.). *n* = 7. The distance signifies the start to the end of the arrow drawn around CAR T cells in the bright-field images in (A). (**D** to **F**) ImageStream flow cytometry for NT and HER2-CAR T cells conjugated with LN229. Nuclei (DAPI), CARs (emerald GFP), actin (phalloidin), LAT, and CTB. Fluorescence intensity of (E) LAT and (F) CTB in immature (non-actin–enriched) versus mature (actin-enriched) CARIS. *N* = 3; *n* = 23,565. (**G**) Schematic representation for isolation of detergent-resistant membranes using detergent iodixanol (OptiPrep) density gradient of CAR T cells (baseline) and CAR T cell tumor cell conjugates, followed by probing fractions for lipid raft (LAT) and nonlipid raft (CD71) markers on WB. Created using Biorender.com. (**H**) WB for membrane fractions from HER2-CAR T cells at baseline (0 min) and in conjugation with LN229 at early (15 min) and late (60 min) CARIS. Probes are for CAR (CD3ζ), phosphorylated CARs (pY142 CD3ζ), LAT (lipid raft marker), and transferrin receptor [CD71, nonlipid raft (non-LR) marker]. Curves show the cholesterol concentration within each fraction. Mwt, molecular weight. (**I** and **J**) Percentage of CAR molecules recruited to the lipid raft fractions of HER2-CAR T cells against LN229 (I) or BT474 (J). Data from three donors pooled [(E) ad (F)], representative of three donors (H), or mean of three donors + SD [(I) ad (J)]. Conditions compared using the Mann-Whitney *U* test with Holm corrected multiple comparisons [(E) and (F)] or RM two-way ANOVA with Holm-Sidak corrected multiple comparisons [(I) and (J)]. **P < 0.01.

The engagement followed by more rapid disengagement of CAR^CD28ζ^ T cells contrasted with the protracted CAR^4-1BBζ^ T cell contact, suggesting that the molecular dynamics at the synapse could be analogously distinct. First, we needed to establish whether mLRs do associate with CARIS as they do with TCR IS (*52*–*63*). We used confocal microscopy to examine the colocalization of emerald green fluorescent protein (emerald GFP)–tagged CAR molecules, with actin, the defining structural protein of the IS; linker for activated T cells (LAT), which molecularly associates with mLRs; and cholera toxin B subunit (CTB), which interacts with the mLR ganglioside GM1 (*70*, *71*). For both HER2-CAR^CD28ζ^ and HER2-CAR^4-1BBζ^, we observed colocalization of CARs, actin, LAT, and CTB at the CARIS (Fig. 3, A to C, and fig. S4, A and B). To substantiate these findings, we confirmed the enrichment of LAT and CTB in mature (actin-enriched) CARIS compared to immature CARIS, using high-throughput flow-based microscopy (ImageStream; Fig. 3D, quantified in Fig. 3, E and F; table S1). Of note, although nontransduced T cells (NT) showed LAT and CTB enrichment at the effector/target contact areas, the intensities of both markers were less than the levels found in CARIS.

Having established the correlation between the CARIS and mLRs, we extracted the mLRs from effector/tumor coculture lysates to sample the protein content of the CARIS. We used high-speed centrifugation (180K to 200K*g*) of a fractionating detergent iodixanol (OptiPrep) density gradient (Fig. 3G). Because mLRs are detergent-resistant membrane fragments, upon centrifugation, they separate, along with their associated proteins, from the detergent fraction and float higher in the fractionation tube by virtue of their higher buoyancy (*72*, *73*). In Western blotting (WB), we defined the mLR fractions based on the presence of LAT and the absence of CD71 (transferrin receptor 1, which only exists outside of mLRs). By this definition, of the nine fractions in our experiments, fractions 1 to 5 contained mLR-associated proteins. To confirm that the isolated fractions correspond to the mLR, we measured the cholesterol concentration, which is enriched in the mLR compartment, and compared the peak cholesterol concentration to LAT+/CD71− fractions, before analyzing lysates for functional proteins. Cholesterol did not completely dissociate from the detergent-rich non-mLR fractions (*6*–*9*). Yet, the trends of cholesterol concentration in mLR fractions (*1*–*5*) were comparable across tested conditions allowing for semiquantitative comparison of mLR proteins (Fig. 3H and fig. S4C). This observation is typical for the adopted mLR isolation protocol, as previously described (*74*).

We compared mLR-associated proteins of the CARIS from HER2-CAR^CD28ζ^ and HER2-CAR^4-1BBζ^ T cells cocultured with HER2-expressing tumor cells (LN229, a nongene-amplified HER2-positive glioma line) at 15 min (early CARIS) and 60 min (late CARIS), against their presynaptic baseline levels (0 min). Our choice of 15 and 60 min is based on the differences observed in our single-cell contact dynamics studies and the patterns of CAR T cell protein phosphorylation previously reported (*75*). In WB, we probed for CD3.ζ and its phosphorylated tyrosine-142 (Y142) at the band that coincided with the CAR molecular weight (51 to 52 kDa; Fig. 3H) (*62*). At baseline (0 min), phosphorylated CAR molecules could be detected in the mLR fractions of CAR^CD28ζ^ T cells more than in CAR^4-1BBζ^ T cells (Fig. 3H, quantified in Fig. 3I). This presynaptic association of CAR^CD28ζ^ with mLRs could help explain the tonic signaling observed with CD28 signaling (*76*). At 15 min of coculture (early CARIS), we observed significant translocation of CAR^CD28ζ^, but not CAR^4-1BBζ^, molecules into the mLR fractions. At 60 min (late CARIS), CAR^CD28ζ^ molecules had returned to presynapse levels, whereas they had accumulated in the CAR^4-1BBζ^-mediated IS mLR fractions. We also observed increased CAR^4-1BBζ^ phosphorylation at 60 min.

When CAR T cells were challenged with antigen-dense tumor targets (BT474, HER2-gene amplified breast cancer cells), a similar and more pronounced pattern of CAR shuttling was observed in CAR^CD28ζ^ and CAR^4-1BBζ^ T cells (Fig. 3J and fig. S4C). The dynamics of CAR^CD28ζ^ engagement to mLRs at a modest antigen density could explain their higher antigen sensitivity when compared to CAR^4-1BBζ^, akin to the reports on the molecular sensitivity of ROR1-CAR^CD28ζ^, cells in response to antigen dose titration (*51*) and functional sensitivity of CD19-CAR^CD28ζ^ T cells in ALL and GPC2-CAR^CD28ζ^ T cells in neuroblastoma (*20*, *23*). The sensitivity of CAR^CD28ζ^ could be explained, at least in part, by its faster and pronounced association with mLRs, contrasting with a slower and limited mLR association with CAR^4-1BBζ^ molecules. When exposed to a high antigen density, more CAR^CD28ζ^ and CAR^4-1BBζ^ molecules interacted with mLRs, suggesting a potential mechanism that explains their enhanced cytotoxicity, and synapse avidity (*77*), when compared to their responses to modest antigen-expressing tumor cells (fig. S4, D and E) (*20*, *23*).

We conclude that the CARIS is associated with mLRs. mLRs interact transiently with CAR^CD28ζ^ molecules in a manner that allows for faster recovery to presynaptic levels. In contrast, the CARIS^4-1BBζ^ showed significant delay in CAR association with mLRs, which could be delaying CAR T cell activation, corresponding to their protracted CARIS formation.

### Activation and recruitment of adhesion molecules to mLRs support the structural avidity of the CARIS^4-1BBζ^

Although the slow and cumulative association of CAR^4-1BBζ^ with mLRs could explain the prolonged attachment of CAR^4-1BBζ^ T cells to target cells, mechanical forces from recruitment of adhesion molecules could also be governing the structural avidity of their CARIS (*40*, *78*). Upon T cell activation, the leukocyte function-associated antigen–1 (LFA-1) undergoes conformational changes that affect its affinity to the intercellular adhesion molecule–1 (ICAM-1) on tumor cells; this is central for the stability of the TCR IS (*79*–*85*).

**Fig. 4.**
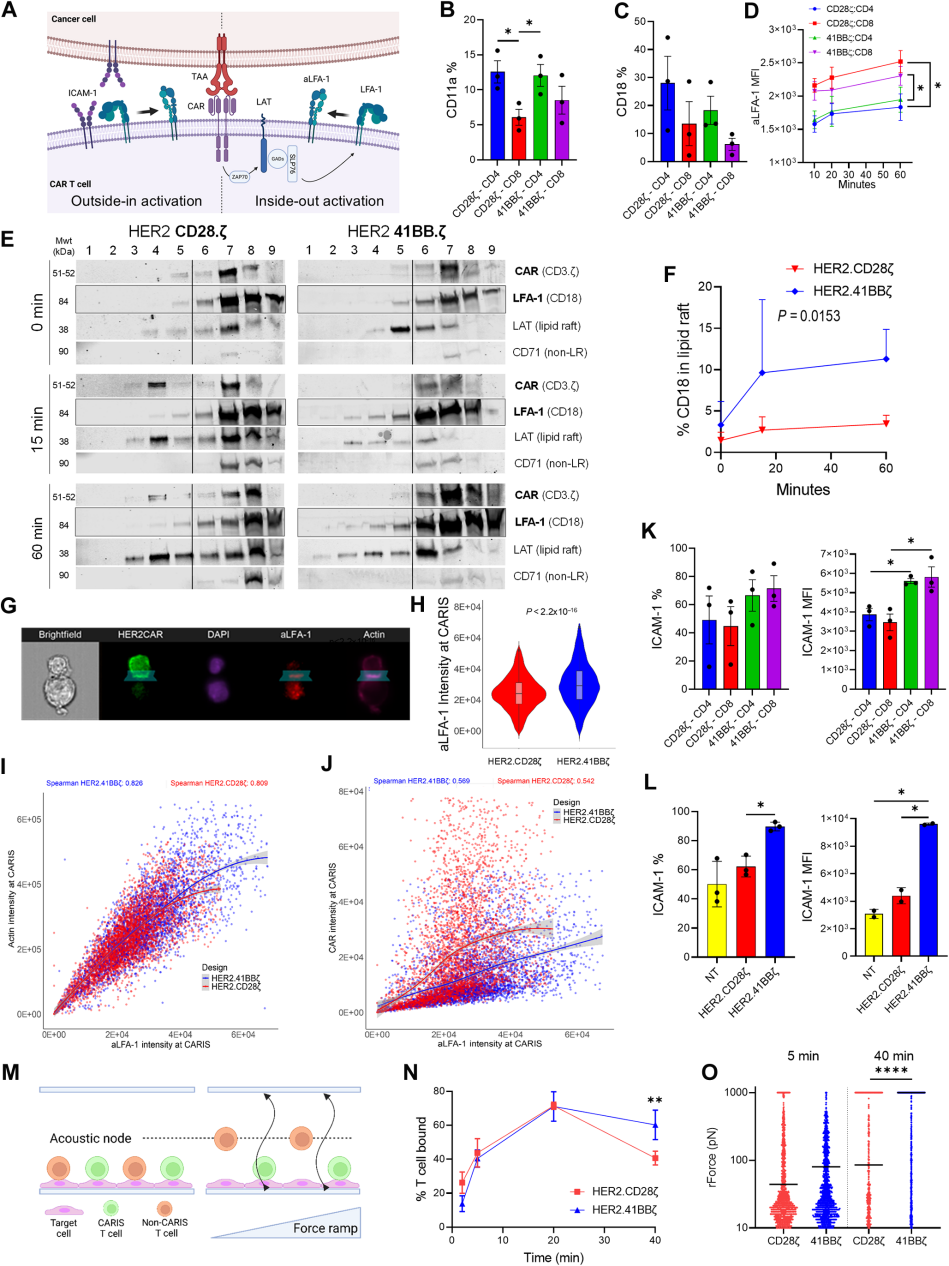
Expression dynamics of adhesion molecules on CAR T cells and the effect on CARIS avidity. (**A**) Hypothetical schematic of mechanisms of LFA-1 activation. (**B** to **D**) Flow cytometry for CD11a (B) and CD18 (C) expression on HER2-CAR T cells at baseline and for aLFA-1 (D) in response to plate bound recombinant HER2, using an LFA-1 extended-open conformation-specific antibody (m24). MFI, mean fluorescence intensity. (**E**) WB for membrane fractions from HER2-CAR T cells at baseline (0 min) and in conjugation with LN229 (15 and 60 min) probed for CAR (CD3ζ), LFA-1 (CD18), LAT, and CD71. (**F**) Percentage LFA-1 (CD18) recruited to the LR fractions from (E). (**G**) ImageStream flow cytometry for HER2-CAR T cells conjugated with LN229 (30 min). CARs (emerald GFP), actin (phalloidin), nuclei (DAPI), and aLFA-1 (m24). (**H** to **J**) Fluorescence intensity of activated aLFA-1 (H) and its correlation with actin intensity (I) or CAR intensity (J) at the CARIS of mature synapses. *N* = 3; *n* = 9007. (**K** and **L**) Flow cytometry for ICAM-1 expression at baseline on CD4 and CD8 (K) and product (L) of HER2-CAR T cells. (**M**) Schematic representation of z-Movi acoustic force microscopy for testing CARIS avidity. A force ramp of 0 to 1000 pN was applied to pull HER2-CAR T cells off an LN229 monolayer toward acoustic nodes. (**N**) z-Movi ultrasound microscopy measuring the percentage of HER2-CAR T cells bound to LN229 at 500 pN after 2, 5, 20, or 40 min of incubation. (**O**) Detachment forces for individual cells after 5 or 40 min of incubation. Median represents the force needed to detach 50% of CAR T cells (EF_50_). Mean of three donors + SD [(B) to (D), (F), (K), and (L)], data pooled from three donors [(H) to (J)] or three to five avidity runs [(N) and (O)] compared using one-way [(B), (C), and (O)] or RM two-way Holm-Sidak corrected ANOVA [(D) and (N), ratio paired *t* test (F), Mann-Whitney *U* test (H), or Spearman correlation (non-Gaussian distribution) [(I) and (J)]. *P < 0.05, **P < 0.01, ****P < 0.0001. (A) and (M) created using Biorender.com.

A role for LFA-1 in the CARIS has been described, but whether it plays a dynamic role in the structural avidity of the CARIS remains undefined (Fig. 4A) (*40*, *44*, *47*). At baseline, we observed higher levels of surface expression of the LFA-1 chains CD11a and CD18 on CD4 compared to their CD8 counterparts of both HER2-CAR^CD28ζ^ and HER2-CAR^4-1BBζ^ T cells (Fig. 4, B and C). To investigate the inside-out activation of LFA-1 upon CAR signaling, we stimulated HER2-CAR T cells on purified plate-bound recombinant HER2 (HER2 Fc) and then assessed for the surface levels of high-affinity LFA-1 using an extended-open conformation-specific antibody (clone: m24) (*86*). HER2 stimulation resulted in gradual unfolding of LFA-1 over time in CD8 T cells and, to a lesser extent, in CD4 T cells (Fig. 4D). We found no significant difference between HER2-CAR^CD28ζ^ and HER2-CAR^4-1BBζ^ T cells.

Next, we investigated the association of LFA-1 with mLRs upon target cell encounter. We observed higher levels of LFA-1 associating with mLRs in both the early (15 min) and late (60 min) CARIS mediated by HER2-CAR^4-1BBζ^ T cells, when compared to HER2-CAR^CD28ζ^ T cells (Fig. 4E, quantified in Fig. 4F). This contrasted sharply with the association pattern of CAR^4-1BBζ^ molecules with mLRs. We then investigated the recruitment of the unfolded/activated LFA-1 (aLFA-1) to the CARIS using flow-based microscopy, ImageStream (Fig. 4G, quantified in Fig. 4, H to J; table S2). At 30 min, HER2-CAR^4-1BBζ^ T cells engaged in more aLFA-1–dense CARIS, compared to CAR^CD28ζ^ T cells. aLFA-1 density at the CARIS^4-1BBζ^ correlated with actin density rather than CAR density at the CARIS. Together, these findings suggest a less CAR-dependent pattern of LFA-1 activation and recruitment to CARIS mLRs in CAR^4-1BBζ^ T cells.

Mamonkin *et al.* reported that a CD5-targeting CAR^4-1BBζ^ was associated with higher expression of ICAM-1, which mediated homotypic cellular interactions (*87*). We observed a comparable finding, where HER2-CAR^4-1BBζ^ T cells expressed significantly higher ICAM-1 when compared to their HER2-CAR^CD28ζ^ counterparts, with no predominant contribution from the CD4 or CD8 compartment (Fig. 4, K and L). Unlike LFA-1, ICAM-1 does not undergo conformational activation (*88*). Accordingly, higher constitutive expression of ICAM-1 on HER2-CAR^4-1BBζ^ T cells would result in an overall higher adhesiveness, which prompted us to perform a cellular avidity assay that can measure the mechanical buildup of adhesive force at the CARIS.

We used the z-Movi Cell Avidity Analyzer (Lumicks, Amsterdam, The Netherlands; Fig. 4M), which assesses the force needed to detach individual CAR T cells from a target monolayer over time using an incremental ultrasound force ramp (*40*). We found that HER2-CAR^CD28ζ^ and HER2-CAR^4-1BBζ^ T cells built up comparable tensile bonds, as measured by percentage of T cells bound at 500 pN of force, after 2, 5, and 20 min of tumor conjugation (Fig. 4N and fig. S4F). At 40 min of tumor conjugation, however, HER2-CAR^CD28ζ^ attachment dropped significantly, whereas HER2-CAR^4-1BBζ^ T cells resisted detachment, needing forces comparable to peak attachment strength. By examining single-cell events, the ramp force needed to detach 50% of T cells (EF_50_) was higher in HER2-CAR^4-1BBζ^ T cells as early as 5 min after tumor conjugation (Fig. 4O), indicating an overall more adherent CARIS. The EF_50_ was significantly higher in CAR^4-1BBζ^ T cells compared to CAR^CD28ζ^ T cells at 40 min, which could explain, in part, why CAR^4-1BBζ^ T cells had protracted attachment to target cells.

We next investigated the contribution of CD4 and CD8 compartments to the differences observed in CARIS avidity. After 5 min of tumor conjugation, more HER2-CAR^4-1BBζ^ CD4 and CD8 T cells were bound to tumor cells after application of detachment forces, when compared to their HER2-CAR^CD28ζ^ T cell counterparts (fig. S4, G and H), and the mean forces for detachment of HER2-CAR^4-1BBζ^ T cells were significantly higher (fig. S4I).

In summary, we observed that CAR^4-1BBζ^ T cells are constitutively more adhesive than CAR^CD28ζ^ T cells, and unlike the latter, they resist detachment from their target cells, which helps explain their longer contact time and lower serial killing capacity.

### Distinct cytolytic mechanisms determine the functional avidity of the CARIS^CD28ζ^

The different patterns of molecular shuttling of CARs and LFA-1 to the CARIS and the associated mechanical buildup could explain the difference in interaction of CAR^CD28ζ^ and CAR^4-1BBζ^ T cells with their target cells. To investigate how these differences affect their functional avidity, namely, their activation and cytotoxic response, we assessed the molecular mechanisms directly used in cell killing.

**Fig. 5.**
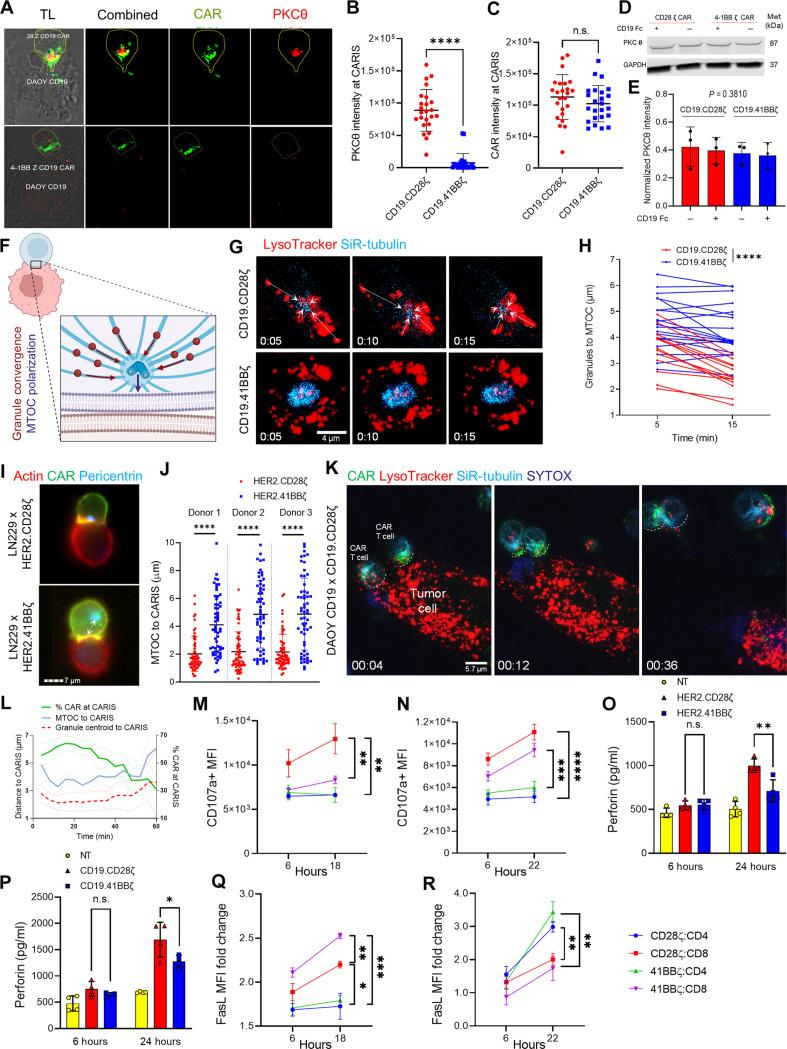
Killing mechanisms deployed by CAR T cells upon tumor cell engagement. (**A**) *Z*-projection confocal microscopy of CD19-CAR T cells conjugated with Daoy.CD19 (20 min), CAR [emerald GFP (green)], and PKCθ (red) and transmitted light (TL). (**B** and **C**) Quantitation of (B) PKCθ and (C) CAR accumulated at CARIS. *n* = 48. (**D** and **E**) WB for total PKCθ in CD19-CAR T cells at baseline and after 20 min of recombinant CD19 stimulation (D), quantified in (E). (**F**) Schematic representation for lytic granule convergence to the MTOC and MTOC polarization to CARIS. Created using Biorender.com. (**G**) Time-lapse imaging of CD19-CAR T cells seeded on CD19 Fc + anti-CD18–coated glass, probed for lytic granules (LysoTracker) and MTOC (SiR-tubulin). (**H**) Change in the mean distance between lytic granules and the MTOC per cell of CD19-CAR^CD28ζ^ (red line) and CD19-CAR^4-1BBζ^ (blue line) T cells. *n* = 32. (**I**) ImageStream for HER2-CAR T cells + LN229 (30 min). CARs [emerald GFP (green)], actin [phalloidin (red)], and MTOC [pericentrin (blue)]. (**J**) Distance between MTOC and CARIS. *n* = 360. Dot, conjugate [(B), (C), and (J)]. (**K** and **L**) Time-lapse tracking of distance of lytic granule centroid (LysoTracker) and MTOC (SiR-tubulin) from CARIS of CD19-CAR^CD28ζ^ T cells + Daoy.CD19 (K) and SYTOX (target cell death); quantification of the percentage of CAR.emeraldGFP at CARIS (green), MTOC distance to CARIS (turquoise), and lytic granule centroid distance to CARIS (red) (L). Mean + SEM. *n* = 15. (**M** and **N**) CD107a flow cytometry on HER2-CAR (M) and CD19-CAR (N) T cells cocultured with LN229 and Daoy.CD19, respectively. (**O** and **P**) Supernatant of HER2-CAR T cells + LN299 (O) and CD19-CAR T cells + Daoy.CD19 (P) tested for perforin using ELISA. (**Q** and **R**) FasL flow cytometry on HER2-CAR (Q) and CD19-CAR (R) T cells cocultured with LN229 and Daoy.CD19, respectively. Student’s *t* test [(B) and (C)], RM two-way ANOVA [(H), (M), (N), (Q), and (R)], or one-way ANOVA [(J), (O) and (P)], with Holm-Sidak corrected multiple comparisons. n.s. = not significant, *P < 0.05, **P < 0.01, ***P < 0.001, ****P < 0.0001.

The early recruitment of the protein kinase C-theta (PKCθ) to the membrane rafts of TCR IS is key to the clustering of downstream signaling complexes, which ultimately leads to the activation of NF-κB (nuclear factor κB), AP1 (activating protein 1), and NFAT signaling prior to the deployment of killing programs (*63*, *89*). We quantified the recruitment of PKCθ to the IS mediated by CD19-CAR^CD28ζ^ and CD19-CAR^4-1BBζ^ T cells at 10 min of CD19 antigen stimulation and 20 min of cell contact (Fig. 5A and fig. S5A). Despite comparable CAR intensity at the CARIS, PKCθ accumulated at significantly higher levels in CARIS^CD28ζ^ (Fig. 5, B and C). This difference was a result of more translocation to the CARIS area rather than a difference in total PKCθ expression levels (Fig. 5, D and E) and is in accord with the reported early Ca^2+^ signaling and phosphorylation patterns of CD19-CAR^CD28ζ^ molecules and more rapid expression of the early activation marker CD69 (*49*, *75*).

Convergence of lytic granules toward the MTOC followed by the polarization of MTOC to the IS (Fig. 5F) is PKCθ dependent and facilitates T cell killing of tumor targets through cytolytic degranulation (*48*, *90*). To investigate granule convergence, we seeded CD19-CAR T cells on glass surfaces coated with CD19 recombinant protein (CD19 Fc) and anti-CD18 (IB4) antibody. Silicon rhodamine tubulin (SiR-tubulin) and LysoTracker were used for live imaging of the MTOC and lytic granules, respectively (*48*). CD19-CAR^CD28ζ^ T cells showed a significant decrease in the mean distance between the granules to MTOC over the course of 15 min of cell contact, when compared to CD19-CAR^4-1BBζ^ T cells, indicating a more rapid granule convergence (Fig. 5G, quantified in Fig. 5H).

To investigate MTOC polarization to the CARIS, HER2-CAR T cells were conjugated to LN229 for 30 min then analyzed using flow-based microscopy, ImageStream. Conjugates with high and comparable actin and CAR densities at the CARIS were selected to measure the distance between the MTOC, stained for pericentrin (*48*), and the CARIS interface. The MTOC was significantly closer to CARIS^CD28ζ^ when compared to CARIS^4-1BBζ^ (Fig. 5I, quantified in Fig. 5J; fig. S5, B and C). By studying the dynamics of MTOC and lytic granule polarization and their relationship to early cytotoxicity in CD19-CAR^CD28ζ^ CAR T cells conjugated with Daoy.CD19, we observed that an increase in CAR density at the CARIS was associated with MTOC and lytic granule polarization. This was reversed at the time of synapse dissociation and target cell death (Fig. 5K, quantified in Fig. 5L; movie S6).

We then investigated the predominant killing program used by CD4 and CD8 T cells from both CAR^CD28ζ^ and CAR^4-1BBζ^ T cells. Specifically, we investigated cytolytic degranulation after tumor encounter by measuring the cumulative surface expression of the lysosomal-associated membrane protein 1 (LAMP-1; CD107a) (*91*). CD107a translocates to the cell membrane with the fusing lytic granule membranes during degranulation. We also probed for secreted perforin and granzyme B from CAR T cells after tumor encounter as a confirmatory measure for cytolytic degranulation. In parallel, we investigated the cumulative expression of Fas ligand (FasL) on the T cell membrane, indicating the likelihood for ligand-based cytotoxicity, after tumor encounter in HER2-CAR T cells (Fig. 5M and fig. S5D) and CD19-CAR T cells (Fig. 5N and fig. S5E). CD8 T cells, especially of CAR^CD28ζ^, were characterized by higher accumulation of CD107a. Although no change in the percentage of cells expressing CD107a occurred over time, an increased molecular intensity per CD107a+ T cell was observed, suggesting repeat degranulation events typical of serial killing. CAR^CD28ζ^ T cells secreted higher levels of perforin, but not granzyme B, in both of the HER2 and CD19 tumor models (Fig. 5, O and P, and fig. S5, F to I). Of note, CD19-CAR^CD28ζ^ T cells showed significantly higher levels of tonic secretion of granzyme B, in the absence of tumor encounter (fig. S5, J and K).

Moreover, a higher antigen density on tumor cells was found to significantly increase CD107a expression on CD8 and CD4 populations of both CAR^CD28ζ^ and CAR^4-1BBζ^ T cells (fig. S5L). On the other hand, HER2-CAR^4-1BBζ^ T cells showed higher accumulation of FasL on the cell membrane, a mechanism that is associated with late killing dynamics (Fig. 5Q and fig. S5D) (*92*). In CD19-CAR T cells, CD4 T cells from both CAR designs showed higher FasL up-regulation, which could be linked to their prolonged contact dynamics (Fig. 5R and fig. S5E).

Collectively, the faster lytic granule convergence, MTOC polarization, and more robust cytolytic degranulation seen with CAR^CD28ζ^ T cells could explain their initially higher functional avidity. On the other hand, the late expression of FasL in CAR^4-1BBζ^ T cells explains why their killing is delayed beyond CARIS formation and why it is relatively sustained over time.

## DISCUSSION

In these studies, we show that the CAR costimulatory endodomain determines the molecular dynamics at the IS and consequently influences the CAR T cell killing behavior. Specifically, CAR^CD28ζ^ molecules rapidly shuttle mLRs mobilizing the MTOC and lytic granules to a CARIS that is transient, acutely lethal, and characterized by higher functional avidity optimal for their serial killing behavior. In contrast, CAR^4-1BBζ^ and LFA-1 accumulate relatively slowly in the mLR, engaging in prolonged CARIS with higher structural avidity but chronic and cooperative target cell killing.

Differences in the behavior of CD28 and 4-1BB signaling could reflect their biological roles in T cells. The immunoglobulin family receptor, CD28, is likely the most effective TCR costimulator as it increases the T cell’s sensitivity to antigens, recruits mLRs to the IS, and mediates cytokine secretion and proliferation (*60*, *93*). Similarly, in CAR T cells, CD28 was linked to robust short-term tumor lysis, heightened sensitivity, and reactivity to modestly expressed, including on-target off-tumor, antigens (*20*, *23*, *51*, *94*). In contrast, the tumor necrosis factor receptor superfamily member 9 (TNFRSF9), widely known as 4-1BB (CD137), is conditionally expressed on activated T cells and promotes T cell survival by inhibiting activation induced cell death (*95*, *96*). In CAR T cells, signaling through 4-1BB mediates mitochondrial biogenesis, persistence, and memory development and an overall more fit CAR T cell, when compared to CD28 signaling (*97*, *98*).

We observed that CD28 mediates short contact and fast killing with the capacity for serial killing. A shorter T^contact^ has been associated with a higher capacity for lymphoma target cell killing and serial killing by CD19-CAR^CD28ζ^ T cells, in vitro (*99*). In a lymphoma model, intravital imaging of CD19-CAR^CD28ζ^ T cells showed that CAR T cells with T^contact^ and T^death^ shorter than 25 min were more lytic (*100*). In our studies, rapid tumor target death coincided with quicker shuttling of CAR^CD28ζ^ to the CARIS. On the other hand, CAR^4-1BBζ^ accumulated slowly at the CARIS interface and resulted in minimal loss of viability in tumor targets even after 60 min of testing. We propose that the prolonged contact CAR^4-1BBζ^ likely compromises their serial killing ability. Yet, it also seems likely that the cytotoxic disadvantage of CAR^4-1BBζ^ could be compensated for, in the long term, by their notable proliferative capacity and their tendency to cooperate (*101*).

We observed, in the context of targeting HER2 and others observed in the context of targeting CD19, that the abundance of CAR^4-1BBζ^ T cells effectors exceeded their anticipated cytolytic effect, indicating that they likely cooperate to kill (*102*). The concept of “additive killing,” described by Weigelin *et al.*, proposes that the killing of more resistant solid tumor cells by T cells can occur with multiple sublethal degranulation doses deposited by multiple T cells that are close enough in time to prevent tumor cell recovery (*101*). T cells were shown to kill leukemia cells using a single hit, which is sublethal in solid cancer cells that require an average of three serial hits (each less than 50 min apart) in a melanoma model. This could support an advantage of CAR^CD28ζ^ signaling in solid cancers. Adapting this concept to our HER2 model, a single HER2-CAR^CD28ζ^ T cell was able to deliver a lethal degranulation dose, making a second hit unnecessary. In contrast, because the HER2-CAR^4-1BBζ^ T cell degranulation is sublethal, subsequent hits were required to produce target cell death, calling for additive or collaborative killing.

It has been suggested that membrane lipid composition influences T cell activation, signaling, and function (*52*–*63*). Specifically, mLRs are thought to provide the necessary rigidity in areas of membrane activity, such as IS formation, IS recruitment of TCRs and associated molecules’ microclusters, and T cell antitumor responses (*52*–*63*). We found CAR^CD28ζ^ to be already within the mLR compartment at baseline, prior to target engagement. A proportion of CAR^CD28ζ^ were already autophosphorylated, which could explain “tonic signaling,” resulting from its clustering (*76*). Upon target engagement, CAR^CD28ζ^ translocate rapidly into and out of the mLR fraction, indicating that the killing process is faster paced. In contrast, the slower accumulation of CAR^4-1BBζ^ molecules coincided with a more prolonged CARIS and consequentially protracted T cell interactions. These differences could be explained by the difference in the biology of CD28 and 4-1BB at mLRs. CD28 was found to play a central role in mediating lipid raft organization. Moreover, CD28 stimulation was found to be central for clustering of signaling molecules, e.g., LCK, PKCθ, and SLP76, at mLRs (*55*, *59*, *60*, *63*). This contrasts with more delayed effects on mLRs upon stimulating 4-1BB (*103*).

mLR association with TCR clusters, also known as TCR patches, has been linked to antigen-independent clustering, which was found to improve antigen detection sensitivity (*104*). Coalescence of preassembled membrane rafts, harboring TCR patches, upon antigen recognition facilitates synapse formation (*105*, *106*). CAR patches have been recently described and compared to TCR patch dynamics and topology (*107*). In these studies, HER2-CAR^4-1BBζ^ patch hyperstability at the IS was associated with altered synapse resolution. Although CAR patch interaction with mLRs was not formally investigated in our studies, our work suggests that preassembled mLRs harboring CAR^CD28ζ^ molecules could explain their faster cytolytic response, compared to the hyperstable CARIS^4-1BBζ^. This is an advantage that CAR^CD28ζ^ may have over CAR^4-1BBζ^ and could be favorable in solid cancers and in scenarios of low target site density. It could also explain the higher toxicity of CD19-CAR^CD28ζ^ T cells observed in the clinic (*5*, *6*, *9*, *10*, *67*).

The slower accumulation of CAR^4-1BBζ^ likely supports its prolonged CARIS, yet the expression and recruitment of adhesion molecules likely mediate this phenomenon. We found that LFA-1 showed higher and sustained inclusion within the mLRs of CAR^4-1BBζ^ T cells upon target engagement, along with the recruitment of more aLFA-1 to the CARIS, regardless of the CAR density at the IS. LFA-1 blockade has been associated with a compromised cytotoxicity of EGFR-CAR^4-1BBζ^ T cells against glioblastoma multiforme (GBM) cells, contrasting with a less critical role in CAR^CD28ζ^ synapses (*40*, *47*). Moreover, CAR^4-1BBζ^ T cells also expressed significantly higher levels of ICAM-1, which is known to undergo heterotypic interaction with LFA-1 and homotypic interaction in *trans* with other ICAM-1 molecules (*88*, *108*, *109*). We previously showed that the homotypic interaction of ICAM-1 on CAR T cells could mediate CAR T cell fratricide (*87*). Here, we therefore suggest that the heterotypic interaction between ICAM-1 and LFA-1, complemented by the homotypic interaction of ICAM-1 in *trans*, can help explain, along with other adhesion axes, the tenacious nature of the synapse mediated by CAR^4-1BBζ^ T cells.

A fast killing pace could stem from the significantly short time between antigen recognition, granule delivery, and target blebbing as described by Davenport *et al.* (*47*, *110*). In our work, CAR^CD28ζ^ induced higher recruitment of PKCθ, faster MTOC polarization, and lytic granule convergence to the CARIS when compared to CAR^4-1BBζ^. CD28 has been found to use diacylglycerol (DAG) as a bait to cluster PKCθ in the TCR IS and boost downstream signaling, which could provide a reasonable explanation for the observed CAR^CD28ζ^ signaling advantage (*111*). DAG also facilitates TCR ectocytosis-mediated IS termination, which could explain their shorter contact time and better serial killing (*112*). We previously described the higher expression of FasL CD19-CAR^4-1BBζ^ at baseline and their robust recruitment to artificial IS, in comparison to CD19-CAR^CD28ζ^ T cells (*113*). In natural killer cells, cytolytic degranulation has been described as an early killing mechanism that is replaced by death receptor–mediated killing over time (*92*). Montalvo *et al.* recently found, in single-cell studies, that blocking granzymes A and B in CD19-CAR^4-1BBζ^ T cells delayed but did not compromise their overall cytotoxicity against ovarian cancer cells (*114*). Blocking of FasL, on the other hand, significantly compromised CD19-CAR^4-1BBζ^ T cell cytotoxicity. In lieu of the aforementioned evidence, the faster killing behavior of CAR^CD28ζ^ T cells could be supported by their fast-paced cytolytic degranulation, whereas CAR^4-1BBζ^ T cells’ accumulation of FasL could explain their protracted killing behavior.

Last, although the mechanism of IS termination remains elusive, it was recently found to be mediated by the cytoskeletal contraction of apoptotic targets (*115*) and could also involve TCR ectocytosis, facilitating serial killing (*112*). In these studies, T cell detachment from contracting target cells was found to be perforin dependent. The delivery of perforin and granzymes drives apoptosis, which entails proteolytic processing of proteins that stabilizes the actin cytoskeleton of the cell (*116*). Actin cytoskeleton is important in maintaining cell-cell contact through an actin-enriched IS (*48*). We have shown that CAR^CD28ζ^ T cells can more rapidly and efficiently undergo lytic granule convergence and induce cytolytic degranulation, evident by more perforin secretion and CD107a accumulation. This could explain CAR^CD28ζ^ T cells’ more efficient detachment from tumor targets.

Molecularly, LFA-1 unfolding associates with IS formation and controls myosin IIA dynamics (*84*, *117*, *118*). It also activates the protein-tyrosine kinases FAK1/PYK1, which clusters with LAT at the expense of its canonical activation cluster: LAT-GAD-SLP-76 complexes, ultimately hampering the T cell’s ability to adhere (*119*). On the other hand, unlike LFA-1, ICAM-1 is a less dynamic molecule that does not undergo conformational changes (*88*). ICAM-1 does mediate IS attachment and signaling (*120*), although its high constitutional expression could help explain the higher structural avidity and delayed detachment in CAR^4-1BBζ^ T cells.

In summary, our studies demonstrate that the CAR signaling domains 4-1BB and CD28 mediate distinct patterns of molecular interaction at the CARIS that affect the single-cell dynamics and consequently the killing behavior of CAR T cells. The understanding of the molecular events at the CARIS interface could help in the rational design of more effective CAR molecules while offsetting their toxicity in the clinic.

## METHODS

### Cell culture, blood donors, and cell lines

CAR T cells were manufactured from peripheral blood mononuclear cells isolated from healthy male and female human donors after informed consent on protocols approved by the Institutional Review Board of Baylor College of Medicine in accordance with the Declaration of Helsinki (protocol #H-45017). CLL-1 is a primary human chronic lymphoblastic leukemia cell line; all other cell lines were purchased from the American Type Culture Collection (Manassas, VA). T cells were maintained in a T cell medium supplemented with IL-7 and IL-15. Human solid tumor cell lines [Daoy (HTB-186), Daoy.2, Daoy.CD19, LN229 (CRL-2611), and BT474 (HTB-20)] and human embryonic kidney (HEK) 293T (CRL-3216/632180; Takeda) were cultured in Dulbecco’s modified Eagle’s medium (DMEM); leukemia and lymphoma cell lines [CLL-1, Nalm6 (G5, CRL-3273), Jeko (CRL-3006), and Raji (CCL-86)] were cultured in RPMI, all supplemented with 1% GlutaMAX and 10 to 20% fetal bovine serum (FBS).

### CAR design and CAR T cell manufacturing

The HER2-specific scFv (FRP5)– and CD19-specific scFv (FMC63)–based CAR transgenes were described previously (*121*, *122*). In brief, CAR transgene design consisted of a target-binding domain scFv connected to a hinge, a transmembrane domain followed by the CD28 or 4-1BB intracellular signaling domain and a ζ signaling domain of the TCR as detailed in the Results section. In some experiments, the CARs have their ζ signaling domain fused to emerald GFP for visualization. All designs were assembled on Clone Manager (Sci-Ed Software, Westminster, CO) or SnapGene software (Dotmatics, Boston, MA), and codon-optimized vectors were synthesized by GeneArt Gene Synthesis service (Invitrogen GeneArt, Regensburg, Germany). Synthetic genes were cloned into an SFG retroviral vector, and the construct confirmed using pyrosequencing (SeqWright DNA-Technology, Houston, TX). Plasmid constructs were amplified in Stellar competent cells (catalog no. 636763; Takara). T cells were transduced with CARs, Daoy were transduced with HER2 (Daoy.2) or CD19 (Daoy.CD19), and Raji were transduced with HER2 (Raji HER2) as described previously (*113*, *121*–*123*).

### Flow cytometry and sorting

Flow cytometry was performed on an Accuri C6 or CANTO II (Becton Dickinson, Franklin Lakes, NJ) flow cytometers. Flow cell sorting was done on BD FACS Aria (Becton Dickinson) or SONY SH800S (Sony Biotechnology, San Jose, CA). Flow cytometry data analysis was performed on the FlowJo analysis software (FlowJo LLC, Ashland, OR). T cell surface expression of FRP5 (HER2-CAR) was detected using conjugated HER2.Fc chimeric protein (catalog no. 1129-ER; R&D Systems, Minneapolis, MN) followed by a fluorophore-conjugated anti-human Fc antibody (catalog no. 12-4998-82; Invitrogen/Thermo Fisher Scientific, Waltham, MA) or using Alexa Fluor 647 (AF647) anti-mouse IgG, F(ab′)2 fragment-specific antibody (catalog no. 715-605-151; Jackson ImmunoResearch Laboratories, West Grove, PA), which was also used for CAR T cell sorting. CD19 expression was detected using a phycoerythrin-or AF647-conjugated anti-FMC63 antibody (catalog nos. FM3-PY54A2 and FM3-AM534; ACROBiosystems, Newark, DE). Direct detection of emerald GFP-fused CARs was also used to detect tagged CARs. HER2 and CD19 antigens were assessed with fluorophore-conjugated specific antibodies (catalog nos. 340554 and 555413; BD). T cell phenotyping was done using anti-CD4 (catalog no. 562971, BD), CD8 (catalog no. 560917, BD), CCR7 (catalog no. 560816, BD), and CD45RA (catalog no. 562885, BD) fluorophore-conjugated antibodies (BD). CD11a (ab52895; Abcam), CD18 (ab185723; Abcam), the conformational m24 antibody (ab13219; Abcam), and ICAM-1 (ab109361; Abcam) were detected by mouse or rabbit anti-human specific antibodies (Abcam, Boston, MA), followed by an AF657 donkey anti-mouse (catalog no. 715-605-151) or AF488 donkey anti-rabbit (catalog no. 711-545-152) specific antibody (Jackson ImmunoResearch Laboratories).

### Impedance-based tumor cell killing assay (xCELLigence)

Long-term tumor cell killing was measured over 160 hours using the xCELLigence (ACEA/Agilent, San Diego, CA). Tumor cells were expanded for 20 to 24 hours in xCELLigence-specific 96-well plates before T cells or Triton X (full lysis) was added. The cell index was monitored every 15 min. A decreasing cell index indicated tumor lysis. The cell index was normalized using the latest reading before adding the T cells.

### Tracking T cell kinetics using long-term time-lapse imaging (Incucyte)

The kinetics of expression of emerald GFP-tagged CAR T cells was tracked for 160 hours using Incucyte S3 (Sartorius, Göttingen, Germany). Non-GFP–tagged tumor cells were expanded for 24 hours prior to adding emerald GFP-tagged CAR T cells, after which plates were added to the Incucyte. Images were captured every 2 hours. Kinetics of the emerald GFP signal were calculated on the Incucyte software and presented as fold change from baseline.

### Time-lapse microscopy

Emerald GFP-labeled CAR T cells were labeled with 5 μM LysoTracker Red DND-99 (Thermo Fisher Scientific) and SiR-tubulin for 30 min at 37°C for centroid and MTOC tracking. Daoy.CD19 cells were labeled with 5 μM SYTOX Blue viability dye (Thermo Fisher Scientific) and calcein AM red (Thermo Fisher Scientific) and then seeded on fibronectin-coated chamber slides (RetroNectin, Takara Bio, San Jose, CA). CAR T cells were added to target cells at a ratio of 1:4 in a final volume of 200 μl of a 1X Opti-Klear solution. Cell mixtures were imaged using a Leica Microsystems SP8 laser scanning confocal microscope with a ×100 magnification, 1.4–numerical aperture (NA) objective. Excitation was provided by an ultraviolet laser at 405 nm and tunable white light laser at 488, 561, and 647 nm. Emission was detected with HyD detectors, and images were collected in a Z stack every 4 min for 120 min. Data were acquired with the LAS AF software (Leica Microsystems, Hesse, Germany) and subsequently exported to the Fiji or Volocity software (PerkinElmer, Waltham, MA) for further analysis.

### Flow cytometry–based cytotoxicity assay

eFluor670-stained leukemia/lymphoma tumor cells were cocultured with emerald GFP-tagged CAR T cells for 24, 48, or 72 hours. After coculture, cells were supplied with 7-aminoactinomycin D (7-AAD) (BD) and counting beads (Invitrogen CountBright Plus) and analyzed on a CANTO II flow cytometer (BD). A constant number of beads were captured across all samples. Absolute numbers of eFluor670+ve emeraldGFP-ve, 7-ADD-ve cells were used to calculate tumor cell viability in comparison to the tumor only controls.

### Time-lapse imaging microscopy in nanowell grids

Polydimethylsiloxane nanowell arrays were fabricated in a petri dish as previously reported (*124*). The array was placed in a plasma chamber for 2 min and quickly hydrated with cell media. Effector and target cells were washed three times with phosphate-buffered saline (PBS) and then labeled with PKH26 Red and PKH67 Green (Sigma-Aldrich, Burlington, MA), respectively. Cells were then washed three times in complete medium and resuspended at a density of 1 million cells/ml. Effector and target cells were loaded onto the nanowell arrays and resuspended in Iscove’s Modified Dulbecco’s Medium (IMDM), 10% FBS, and annexin V AF647 (Invitrogen). The wells were imaged using bright-field, AF488, Texas Red, and Cy5 filters. We used a Carl Zeiss Axio Observer microscope (ZEISS AG, Oberkochen, Germany) fitted with a Hamamatsu Orca-Flash sCMOS camera (Hamamatsu Photonics, Bridgewater, NJ) and a ×20, 0.8-NA objective and imaged for 6 hours every 5 min. Cell tracking and quantification were performed as described previously (*64*).

Effectors and targets are mixed at a 1:1 ratio before adding them to the nanowell grid. For contact dynamics, wells that have 1 effector and 1 target are selected for analysis. For studying serial killing, wells that have 1 effector and 2+ targets are selected for analysis. For studying cooperative killing, wells that has 2+ effectors and 1 target are selected for analysis.

### Multiplex cytokine analysis (MILLIPLEX cytokine assay)

Supernatants from HER2 and CD19-CAR T cells cocultured with LN229 and Daoy.CD19, respectively, were collected after 24 and 48 hours, and cytokines were quantified using the MILLIPLEX Human T cell High Sensitivity 13-Plex Panel [GM-CSF, IFN-γ, IL-1β, IL-2, IL-4, IL-5, IL-6, IL-7, IL-8, IL-10, IL-12 (p70), IL-13, and TNF-α] according to the manufacturer’s instructions (R&D Systems). Data were acquired on a Luminex xMAP instrument (Luminex Corporate, Austin, TX).

### Confocal microscopy

Emerald GFP-labeled CAR T cells were mixed with target cells in 200 μl of T cell media and incubated for 10 min at 37°C. The cell mixture was then gently transferred to silane-coated glass slides (Electron Microscopy Sciences, Hatfield, PA) and incubated for an additional 10 min at 37°C. The slides were then washed and permeabilized and fixed with a BD Perm/Fix solution (BD). In the case of CTB staining, CTB was diluted 1:1000 in DMEM and added to the slide for 30 min at 4°C prior to permeabilization. The reagents/antibodies used were as follows: anti-human LAT (CST), AF647 CTB (Invitrogen, Carlsbad, CA), AF568-conjugated phalloidin (Life Technologies, Carlsbad, CA), anti-pericentrin rabbit polyclonal antibody (Abcam), AF647-conjugated mouse anti-perforin clone δG9 (BD), and anti-PKCθ antibody (Santa Cruz Biotechnologies, Dallas, TX). Slides were mounted with 0.15-mm coverslips (VWR, Radnor, PA) using ProLong Antifade (Invitrogen) or Vectashield Antifade mounting media (Vector Labs, Newark, CA). Images were acquired using similar settings as described in live-cell confocal microscopy.

### Imaging flow cytometry (ImageStream)

T cells (2 × 10^6^ per condition) were cocultured with LN229-GBM cells (1:1 ratio) in DMEM with 10% FBS and 1x GlutaMAX. Cells were collected and fixed with Cytofix (BD Biosciences) followed by a BD Phosflow Perm/Wash Buffer (BD Biosciences), except for CTB, which was added for 30 min at 4°C before fixation. Cells were then stained with [LAT, CTB, and aLFA-1 (m24)], 4′,6-diamidino-2-phenylindole (DAPI) (20 μg; D9542-10MG; MilliporeSigma, Burlington, MA), and phalloidin conjugated to AF750 (1 μl; Thermo Fisher Scientific) and resuspended in 50 to 100 μl of a permeabilization buffer. Samples were acquired using ImageStream Mk II (Amnis, Seattle, WA), with a ×60 objective, and >30,000 events with a root mean square of >0.5 were acquired using ISX. Compensation datasets were acquired under similar illumination conditions using single stain samples. Data were analyzed using IDEAS 6.0 (Amnis). Event capture and gating strategy for CAR+ T cell and GBM conjugates are detailed in fig. S6.

### Lipid raft isolation and cholesterol quantification

T cells (100 × 10^6^ to 150 × 10^6^) in 5 ml, tumor target cells (100 × 10^6^ to 150 × 10^6^) in 5 ml, or T cells (100 × 10^6^ to 150 × 10^6^) mixed with target cells (100 × 10^6^ to 150 × 10^6^) at 1:1 ratio in 10 ml in a 50-ml polystyrene conical tube were centrifuged at 370*g* for 1 min and were processed directly (baseline) or incubated in 5% CO_2_ at 37°C for 15 or 60 min. Cells were then spun down (5 min at 400*g*), and the pellet was resuspended in 5 ml of an ice-cold TNE (10 mM tris base, 100 mM NaCl, and 1 mM EDTA) buffer (Quality Biological Inc., Gaithersburg, MD), then centrifuged (5 min at 400*g*), and resuspended in 1 ml of ice-cold TNE + 10 μl of protease inhibitor cocktail (100x) (MilliporeSigma) and 10 μl of phosphatase inhibitor cocktail (100x) (MilliporeSigma). Cells were homogenized 15 times by passing through a 27-gauge needle (BD). The lysate was spun down (5 min at 1000 to 1250*g* at 4°C). Then, 900 μl of the homogenate supernatant was collected in a clean ultraclear centrifugation tube (Beckman Ultra-Clear tubes, 344060, 14 ml, 14 mm by 95 mm, Beckman Coulter). From this point on, all subsequent steps were performed at <4°C. One hundred microliters of ice-cold 10% TX-100 (Triton X-100 Surfact-Amps, Thermo Fisher Scientific) was added to the homogenate supernatant and left on ice for 30 min. All 1000 μl of detergent-extracted samples was brought to 40% iodixanol by adding 2000 μl of OptiPrep (60% iodixanol; Sigma-Aldrich) and then overlaid with layers of descending concentration of iodixanol, diluted in a TNE buffer, using the following order: 2000 μl of 30% iodixanol, 2000 μl of 25% iodixanol, 2000 μl of 15% iodixanol, 2000 μl of 10% iodixanol, and then 2000 μl of 5% iodixanol.

All overlays were added dropwise alongside of the tube, avoiding mixing of layers, and then centrifuged at 37K to 40K rpm (180K to 200K*g*) for 15 to 18 hours at 4°C (Beckman Coulter SW40 rotor). After centrifugation, nine fractions of 1446 μl each from top were collected, and then 25 to 50 μl of each fraction was used to measure cholesterol concentration using the Amplex Red Cholesterol Assay Kit (Invitrogen) read on a BioTek Synergy H4 Plate Reader (Agilent, Santa Clara, CA). Samples of 30 μl of each fraction were mixed with 10 μl of a 4x reducing Laemmli buffer (Bio-Rad), boiled for 10 min at 95°C, and then loaded on 12% precast polyacrylamide gel (Bio-Rad).

### Western blot

Cell lysates from an equal number of T cells from each experimental condition were treated using a radioimmunoprecipitation assay lysis and extraction buffer (Thermo Fisher Scientific) supplemented with protease inhibitor cocktail set III, EDTA-Free (MilliporeSigma). Lysates were heated for 10 min at 95°C with a Laemmli buffer (Bio-Rad, Hercules, CA) supplemented with 2-mercaptoethanol (Bio-Rad) and loaded on 12% Mini-PROTEAN TGX Stain-Free Protein Gels (Bio-Rad) against Amersham ECL rainbow molecular weight markers, full range (GE HealthCare) in a Mini-PROTEAN Tetra Vertical Electrophoresis Cell (Bio-Rad). Protein was then transferred to nitrocellulose (NC) membranes compatible with the iBlot 2 Transfer system (Life Technologies). NC membranes were blocked in 5% nonfat milk in a tris-buffered saline with Tween 20 (TBST) buffer (Bio-Rad) for 1 hour. Primary antibodies against proteins of interest were probed along with glyceraldehyde-3-phosphate dehydrogenase (GAPDH) (loading control) (Cell Signaling Technologies, Danvers, MA) at dilutions ranging from 1:100 to 1:1000 in 5% nonfat milk in a TBST buffer overnight at 4°C. Secondary antibodies coupled to infrared or near-infrared fluorescent dyes “IRDye” (catalog nos. 925-32210 and 925-68071; LI-COR, Lincoln, NE) diluted at 1:5000 to 1:10,000 in TBST with species specificity against the primary antibodies were used for protein detection. NC membranes were then imaged using an Odyssey CLx (LI-COR). Images were analyzed using Image Studio (LI-COR) and Fiji.

Details of primary antibodies used in WB: CAR (CD3ζ; clone: 6B10.2; catalog no. sc-1239; Santa Cruz Biotechnologies), phosphoCAR [CD3ζ-phosphotyrosine Y142; clone: K25-407.69 (RUO); catalog no. 558402; BD Biosciences], LAT (clone: E3U6J; catalog no. 45533S; Cell Signaling Technologies), transferrin receptor (CD71; clone: 3B8 2A1; catalog no. sc-32272; Santa Cruz Biotechnologies); LFA-1 (CD18; catalog no. Ab185723; Abcam), PKCθ (clone: E1I7Y; catalog no. 13643; Cell Signaling Technologies), and GAPDH (clone: 14C10; catalog no. 2118; Cell Signaling Technologies).

### CARIS avidity (z-Movi)

Experiments for CARIS avidity measurement were done on an ultrasound microscope (z-Movi, Lumicks, The Netherlands) were performed according to the standard protocol. Briefly, z-Movi chips were coated with poly-l-lysine (Sigma-Aldrich, catalog no. P4707) for 5 min, dried, rehydrated with PBS, and then conditioned with complete DMEM. Avidity measurements were conducted on a z-Movi Cell Avidity Analyzer, controlled by a computer running the proprietary Oceon software. Using a 3-ml Luer Lock Syringe (Terumo, catalog no. SS+03L1) to create negative pressure in the z-Movi chip flow channel, LN229 cells (50 × 10^6^ cells/ml) were seeded onto z-Movi chips, creating a monolayer. The z-Movi chip was then sealed and incubated in a dry incubator for 30 min prior to media change and an additional 2-hour incubation. During this time, T cells were collected, washed in PBS, stained with CellTrace Far Red (Thermo Fisher Scientific, catalog no. C34564) for 15 min and washed again before being resuspended in T cell media at 5 × 10^6^ cells/ml. Before the first experiment on each z-Movi chip, the LN229 monolayer was validated by applying force for 10 s followed by washing, removing dead and unattached cells. Fluorescently labeled T cells were flowed onto the monolayer such that 200 to 500 cells were in the field of view. T cells were then incubated with the target cell monolayer for the indicated time (2, 5, 20, or 40 min) before the start of the force ramp. Force ramp was set at 1000 pN over 2.5 min for each run. After each force ramp, detached cells were washed away, and then the next sample of effector cells was flowed on.

Avidity experiments were processed using the proprietary Oceon software. Briefly, bright-field and fluorescence preflow images and time-lapse movies were loaded in Oceon; fluorescently labeled cells already present at the start of imaging (“stuck cells”) were automatically detected and removed. Using the first frame of the time-lapse movies, flowed-in effector cells were automatically detected. Those at the edge of the field of view, not forming an interface with one or more target cells (“glass cells”), or in contact with one or more effector cells (doublets/clusters), were manually removed. The remaining cells were then analyzed for displacement over the course of the force ramp. At the end of the time-lapse, the categorization of each cell was manually validated; cells that remained attached to target cells but moved within 1 cell diameter of their original position were reclassified as “hinge” cells, and cells whose original position was obscured by detached cells clustered at the acoustic node at the end of the force ramp were excluded from analysis.

### Test for T cell mechanism of killing

T cells only or T cells cocultured with tumor targets were supplemented with Allophycocyanin (APC) anti-human LAMP-1/CD107a (BD), anti-CD178/FasL (BioLegend), and 1% protein transport inhibitor (containing monensin) (BD) in the coculture media and incubated in 96-well plates for 6, 18, or 22 hours. Cells were collected and washed before staining for CD4 and CD8 markers. Cells were analyzed on BD Canto II flow cytometer.

### Perforin and granzyme B detection using ELISA and ELISpot assay

For enzyme-linked immunosorbent assay (ELISA), CAR T cells (1 × 10^5^) were cocultured with tumor cells (2 × 10^5^) in 200 μl of complete media in 96-well flat-bottom tissue culture plates. At 6- and 24-hour time points, plates were centrifuged at 400*g* for 5 min, and supernatants were collected. The levels of perforin and granzyme B were quantified using protein-specific ELISA kits (Invitrogen, catalog nos. BMS2306 and BMS2027-2) according to the manufacturer’s protocol.

Enzyme-linked immunospot (ELISpot) analysis was used to quantitate the frequency of granzyme B–secreting cells. Briefly, tumor cells were resuspended at 1 × 10^6^ cells/ml in a cell culture medium and 100 μl of cells was added to each ELISpot well. Then, T cells (CAR or nontransduced T cells) were resuspended at 0.05 × 10^6^ cells/ml and 100 μl was added to the tumor cells in the ELISpot wells. Antigen-specific activity was measured after stimulation of T cells with their cognate tumor cell targets, whereas tumor cells alone, CAR T cells, and nontransduced T cells alone were used as controls. After 16 to 18 hours of incubation, plates were developed, dried overnight at room temperature, and then quantified using the IRIS ELISpot/FluoroSpot reader (Mabtech Inc., Cincinnati, OH). Spot-forming cells and input cell numbers were plotted accordingly.

### Statistical analysis

Data were analyzed in the Prism v8 software (GraphPad, La Jolla, CA) and presented as means ± SD unless annotated otherwise in figure legends. Analysis of variance (ANOVA) and Student’s *t* test were accompanied with Holm-Sidak correction for multiple pairwise comparisons. Survival analyses (Kaplan-Meier) were tested using both log-rank and Mantel-Cox tests. Categorical analyses were tested using χ^2^ tests. Testing for data distribution was done for high-throughput data. Data that did not follow Gaussian distribution were analyzed using nonparametric tests because there is less of a possibility to reach incorrect conclusions using Kruskal-Wallis with Dunn’s correction for multiple comparisons. For high-throughput data, for example, ImageStream microscopy single-cell data, R was used for statistical analysis. Single data frame was generated from each donor points to streamline the downstream analysis. To visualize the data distribution, we used histogram and Q-Q plots to qualitatively assess data normality. The interquartile range method was used to remove outliers where it was performed individually on each CAR design and the corresponding raw data. The Shapiro-Wilk test was used to test for normality when the datasets have less than 5000 inputs, whereas the Anderson-Darling test was used when the data had more than 5000 data inputs. Violin plots were created to display the overall distribution and central tendency of each dataset. Because of non-Gaussian data distribution, the Mann-Whitney *U* test, with Holm correction for multiple pairwise analyses, was computed to analyze the statistical significance between groups. In addition, Spearman’s rank correlation was calculated to test the relationship between groups. R packages used include ggplot2, dplyr, tidyr, and reshape2. The R codes generated are provided as data S1 and S2.

A *P* value of <0.05 was considered significant, and the threshold of significance is denoted by n.s., not significant, *P < 0.05, **P < 0.01, ***P < 0.001, and ****P < 0.0001. Statistical tests used for each experiment are mentioned in the figure legends.

### Graphic design and video editing

All graphical illustrations in Figs. 1, E and L, 2E, 3G, 4, A and M, and 5F have been designed on Biorender.com. Video editing, labeling, and collage were done on Microsoft Clipchamp.
