# Supplementary material for: Molecular dynamics at immune synapse lipid rafts influence the cytolytic behavior of CAR T cells
Source: Sci Adv. 2025 Jan 10;11(2):eadq8114. doi: 10.1126/sciadv.adq8114 (PMC11721525; doi:10.1126/sciadv.adq8114)
Supplement: Supplementary file 1 — Figs. S1 to S6 Legends for movies S1 to S6 Legends for tables S1 and S2 Data S1 and S2 [file sciadv.adq8114_sm.pdf]

Science Advances
AAAS

Supplementary Materials for

Molecular dynamics at immune synapse lipid rafts influence the cytolytic behavior of CAR T cells

Ahmed Z. Gad *et al.*

Corresponding author: Nabil Ahmed, nabil.ahmed@bcm.edu

Sci. Adv. **11**, eadq8114 (2025)
DOI: 10.1126/sciadv.adq8114

The PDF file includes:

Figs. S1 to S6
Legends for movies S1 to S6
Legends for tables S1 and S2
Data S1 and S2

Other Supplementary Material for this manuscript includes the following:

Movies S1 to S6
Tables S1 and S2

**List of Supplementary Materials:**
Supplementary Figures 1-6
Supplementary Videos 1-6
Supplementary Tables 1-2
Supplementary Data 1-2

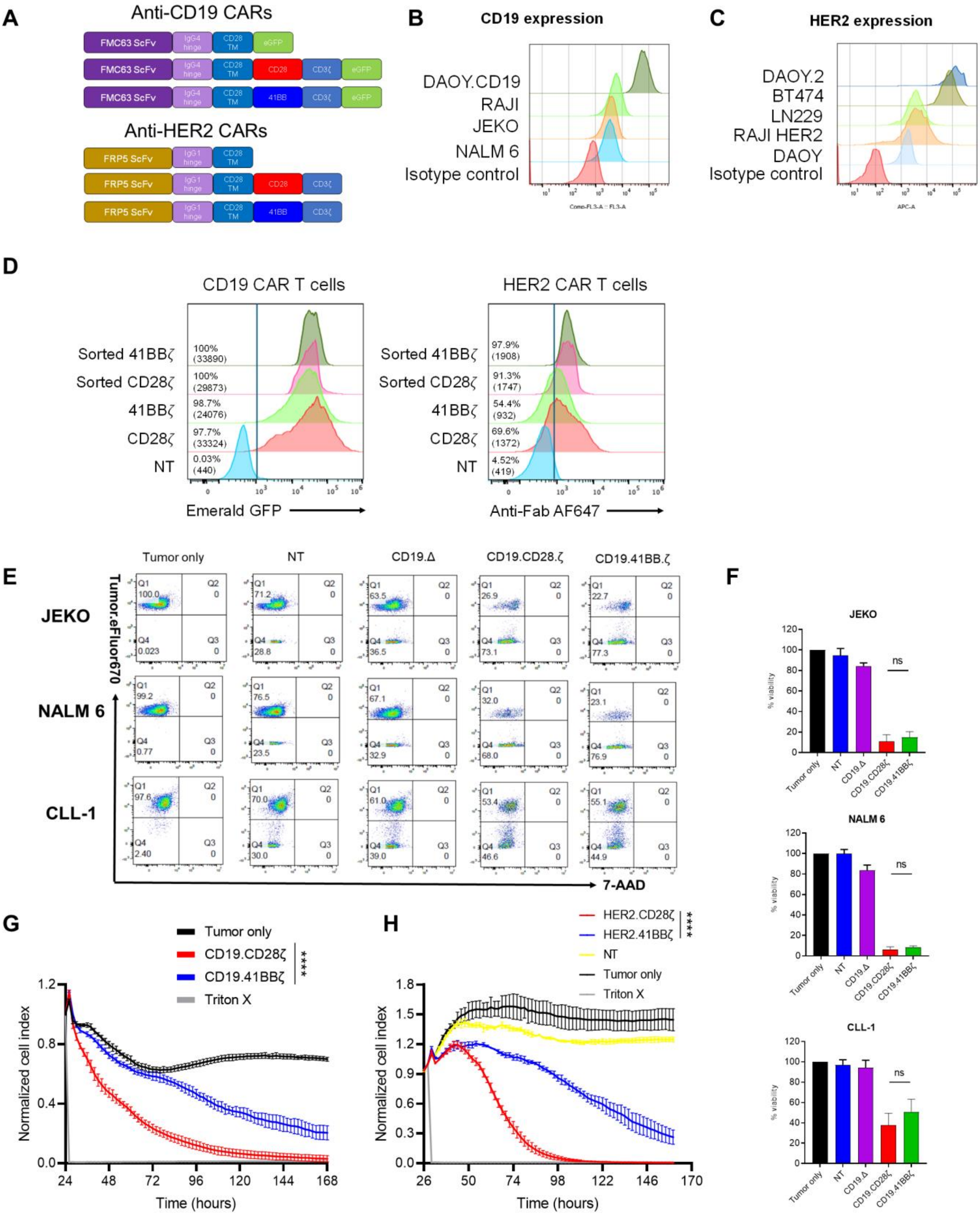
A
Anti-CD19 CARs
FMC63 ScFv
IgG4 hinge
CD28 TM
eGFP
CD28
CD3ζ
41BB
Anti-HER2 CARs
FRP5 ScFv
IgG1 hinge
B
CD19 expression
DAOY.CD19
RAJI
JEKO
NALM 6
Isotype control
C
HER2 expression
DAOY.2
BT474
LN229
RAJI HER2
DAOY
D
CD19 CAR T cells
HER2 CAR T cells
Sorted 41BBζ
Sorted CD28ζ
41BBζ
CD28ζ
NT
Emerald GFP
Anti-Fab AF647
E
Tumor only
NT
CD19.Δ
CD19.CD28.ζ
CD19.41BB.ζ
JEKO
NALM 6
CLL-1
Tumor.eFluor670
7-AAD
F
% viability
ns
G
Tumor only
CD19.CD28ζ
CD19.41BBζ
Triton X
Normalized cell index
Time (hours)
H
HER2.CD28ζ
HER2.41BBζ
NT
Tumor only
Triton X

Supplementary Figure 1: Cytotoxicity and expansion of CAR T cells upon target encounter. A. Design of CD19-CAR and HER2-CAR molecules having CD28 transmembrane domain (TM). TM could influence the CAR reactivity threshold and CARIS efficiency (*23*), accordingly we chose CD28 TM, over CD8α TM, across the CD19- $CAR^{4\text{-}1BB\zeta}$ and $CAR^{CD28\zeta}$, for molecular live-cell tracking, and fused eGFP to CAR signaling domains. **B.** Flow-cytometry for CD19 expression on tumor cell lines used through this study. **C.** Flow-cytometry for HER2 expression on tumor cell lines used through this study. **D.** HER2- and CD19-CAR T cells were flow sorted before functional assays to obtain similar transduction rates and intensities. CD19-CAR T cells were sorted on the emerald GFP+ tag in CARs (left) or using an anti-FMC63 antibody labelled with AlexaFlour647 for untagged CARs. HER2-CAR T cells were sorted on the emerald GFP+ tag in CARs or using an anti-Fab antibody labelled with AlexaFlour647 for untagged CARs (right). **E.** Flow-cytometry based cytotoxicity assay. CD19-CAR T cells were cocultured with JEKO, Nalm-6, and primary CLL (CLL-1) tumor cells. Tumor cells were stained with eFlour670. 7-ADD was used as an exclusion apoptotic marker while counting viable tumor cells, quantified in (**F**). **G-H.** In xCELLigence electrical impedance cytotoxicity assays, Daoy.CD19 cells were incubated for 24-26 hours, then CD19- (**G**) or HER2- (**H**) CAR T cells were added at an E:T seeding ratio of 1:5. A decrease in normalized cell index indicates a decrease in residual Daoy.CD19 cells. Triton-X was used as a full tumor cell lysis control. Statistical comparisons were done on areas under the curve (AUC). ANOVA with Holm-Sidak correction for pairwise multiple comparisons was used (F, G and H). Mean of 3 donors (F) or representative of 3-5 donors (D, E, G, and H).

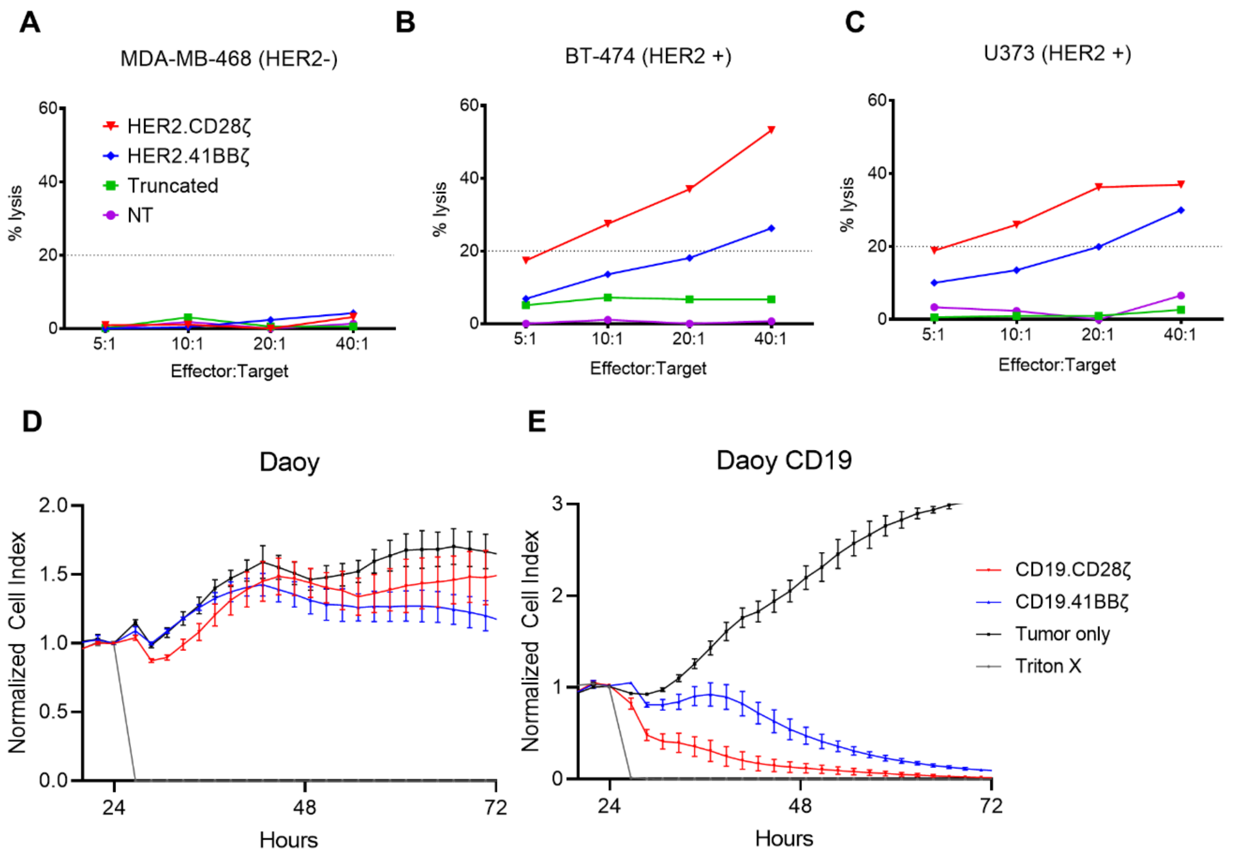

<u>**Supplementary Figure 2**</u>**: Test for short-term allo-reactivity of HER2-CAR T cells and long-term allo-reactivity of CD19-CAR T cells. A-C.** Tumor cells were incubated in Chromium-51 for 1 hour before co-culture with HER2-CAR T cells for 4 hours. Supernatants of MDA-MB-468 (**A**), BT-474 (**B**), and U373 (**C**) were measured for the levels of radioactivity. The 20% line represents the release cutoff for clinical products. **D-E.** CD19-CAR T cells were added to WT Daoy (**D**) or Daoy.CD19 (**E**), 24 hours after tumor growth at an E:T seeding ratio of 1:5. Representative of 2 donors tested (A-E).

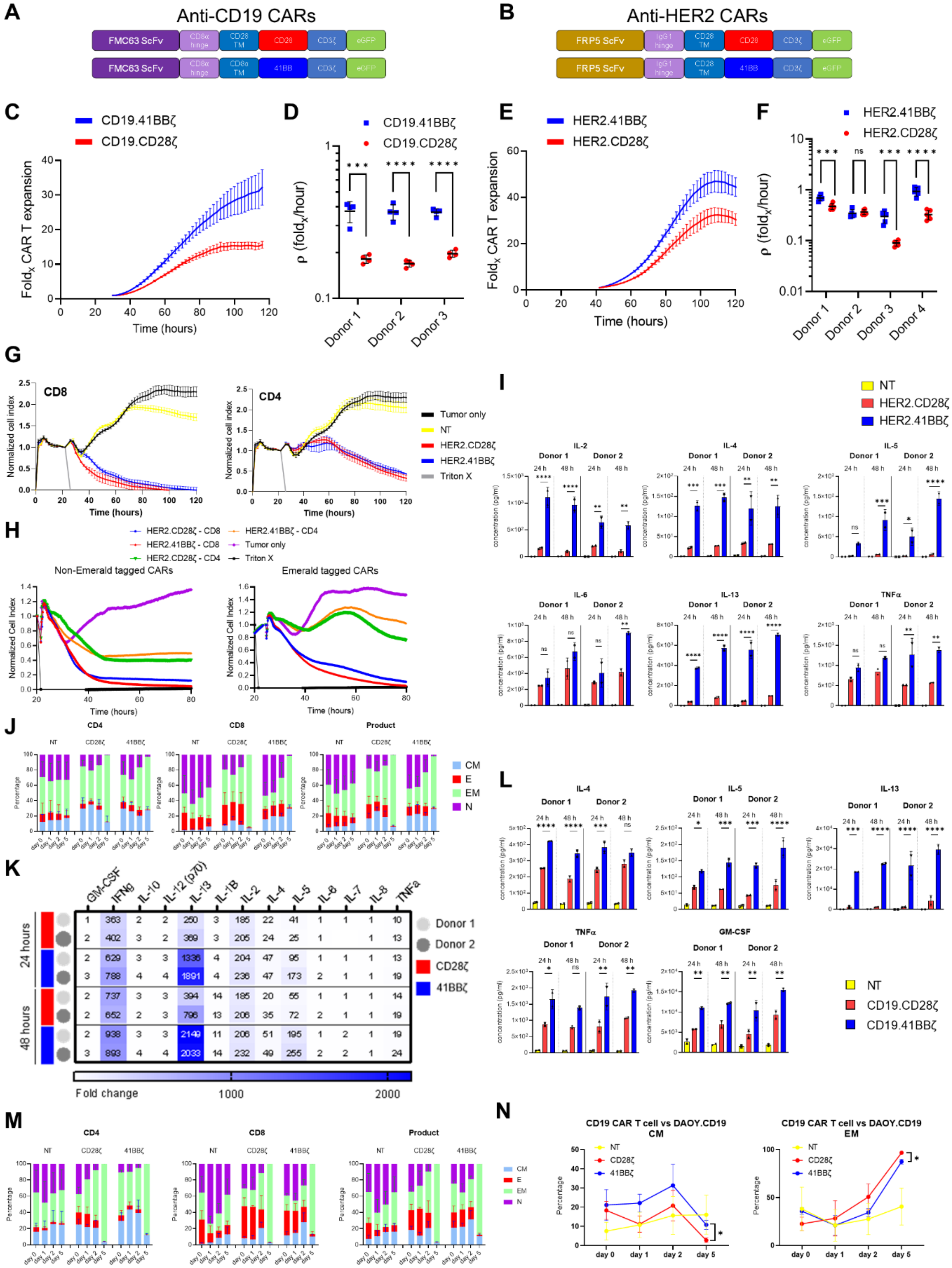
A
Anti-CD19 CARs
FMC63 ScFv
CD8α hinge
CD28 TM
CD28
CD3ζ
eGFP
CD8α TM
41BB
B
Anti-HER2 CARs
FRP5 ScFv
IgG1 hinge
C
CD19.41BBζ
CD19.CD28ζ
Foldx CAR T expansion
Time (hours)
D
ρ (foldx/hour)
Donor 1
Donor 2
Donor 3
E
HER2.41BBζ
HER2.CD28ζ
F
Donor 4
ns
G
CD8
CD4
Normalized cell index
Tumor only
NT
Triton X
H
HER2.CD28ζ - CD8
HER2.41BBζ - CD8
HER2.CD28ζ - CD4
HER2.41BBζ - CD4
Tumor only
Triton X
Non-Emerald tagged CARs
Emerald tagged CARs
Normalized Cell Index
I
IL-2
IL-4
IL-5
IL-6
IL-13
TNFα
24 h
48 h
concentration (pg/ml)
J
Product
CM
E
EM
N
Percentage
K
GM-CSF
IFNg
IL-10
IL-12 (p70)
IL-1B
IL-8
IL-7
24 hours
48 hours
Donor 1
Donor 2
CD28ζ
41BBζ
Fold change
1000
2000
L
GM-CSF
CD19.CD28ζ
CD19.41BBζ
M
N
CD19 CAR T cell vs DAOY.CD19 CM
CD19 CAR T cell vs DAOY.CD19 EM
day 0
day 1
day 2
day 5

<u>Supplementary Figure 3</u>: Characterization and functional analysis of CAR T cell subsets. **A-B.** Design of FDA-approved CD19-CAR molecules (**A**) and HER2-CAR molecules (**B**) tagged with eGFP. **C-F.** Expansion using Incucyte time-lapse imaging of (**C**) CD19-CAR T cells (ρ quantified in **D**) and (**E**) HER2-CAR T cells (ρ quantified in **F**) in response to Daoy.CD19 and LN229, respectively. The proliferative capacity of T cells upon tumor encounter quantified by adopting a mathematical equation described by *Stein et. al.* (*29*). In this equation, a CAR T cell expansion rate constant (ρ) is calculated using the fold expansion of CAR T cells ($fold_x$) from baseline to the time (T_{max}) of peak expansion (C_{max}): $\rho = \log(fold_x)/T_{max}$. **G-H.** xCELLigence cytotoxicity assay for CD8 and CD4 HER2-CAR T cell added at a 1:5 ratios of LN229 seeding density. The tumor-only control is the same on both graphs since it is a single experiment, yet CD4 and CD8 were separated on two graphs for visual clarity (**G**). LN229 tumor cells were incubated 20 hours prior adding non-Emerald tagged CAR T cells (sorted on CD4 or CD8) (left) or Emerald tagged CAR T cells (sorted on CD4 or CD8) (right) (**H**). **I.** Cytokine concentrations from multiplex (MILLIPLEX) assay done on the supernatant collected 24 and 48 hours from HER2-CAR T cells (50/50 CD4:CD8) cocultured with LN229. **J.** CCR7 and CD45RA were probed to track the change in HER2-CAR T cell memory phenotype: Naïve (N), effector memory (EM), effect (E), and central memory (CM) after being cocultured with LN229. **K-L.** Cytokine multiplex (MILLIPLEX) done on the supernatant collected 24 and 48 hours from CD19-CAR T cells (50/50 CD4:CD8) cocultured with Daoy.CD19, values represent mean from duplicates for fold change of the NT of the same condition (**K**) and cytokine concentration from multiplex assay (**L**). **M.** CCR7 and CD45RA were probed to track the change in CD19-CAR T cell memory phenotype exposed to Daoy.CD19: Naïve (N), effector memory (EM), effector (E), and central memory (CM). **N.** Change in EM and CM percentages of CD19-CAR T cells. One-way ANOVA (D, F, I and L) or RM two-way ANOVA (N) with Holm-Sidak correction for pairwise multiple comparisons.

The data is representative of 3-4 donors (C, E, G, and H) or the mean and SD for 3 healthy donors (J, M and N).

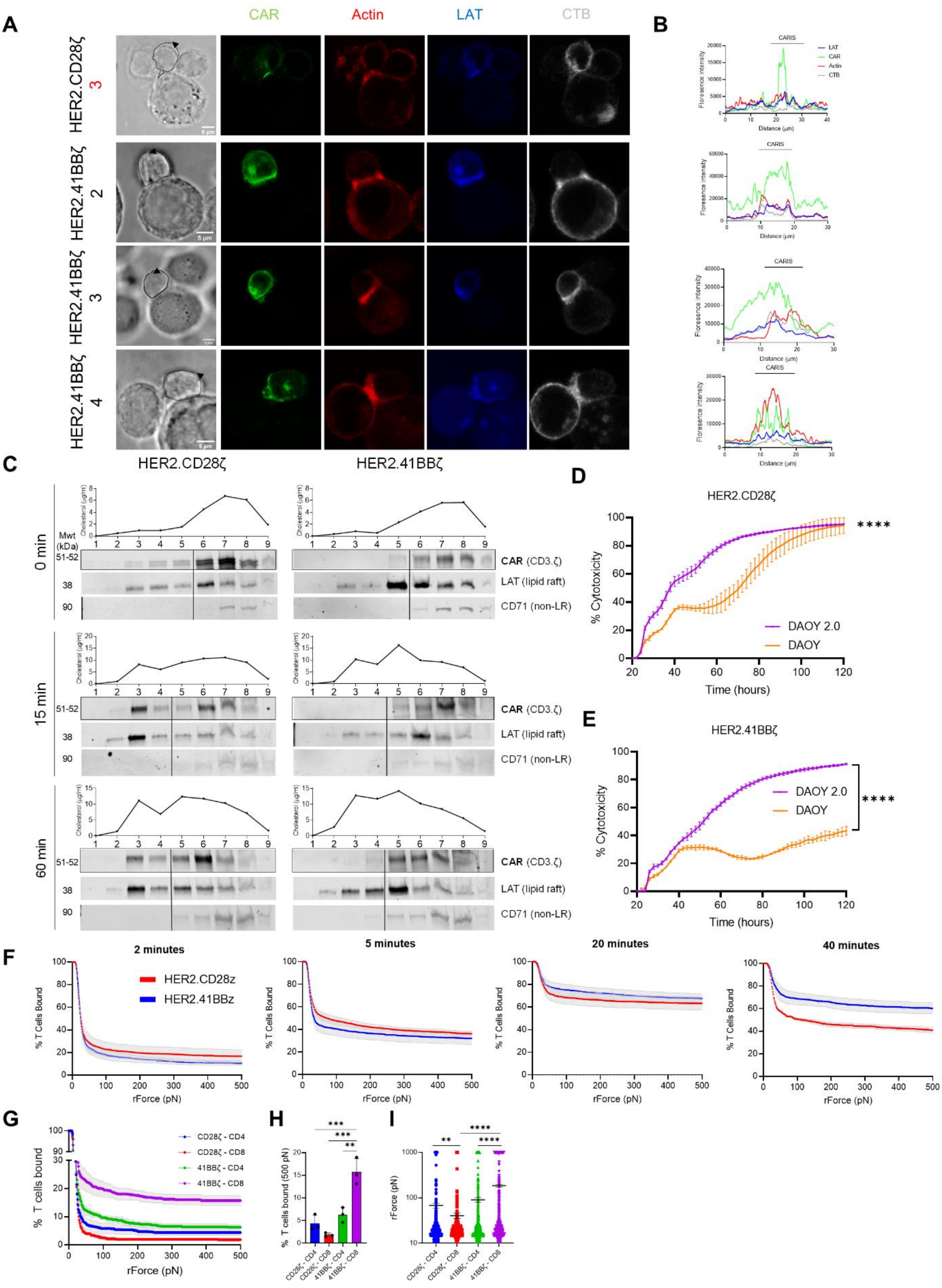

A
CAR
Actin
LAT
CTB
HER2.CD28ζ 3
HER2.41BBζ 2
HER2.41BBζ 3
HER2.41BBζ 4
5 µm
B
CARIS
LAT
CAR
Actin
CTB
Floresence intensity
Distance (µm)
C
HER2.CD28ζ
HER2.41BBζ
0 min
15 min
60 min
Mwt (kDa)
51-52
38
90
Cholesterol (µg/ml)
CAR (CD3.ζ)
LAT (lipid raft)
CD71 (non-LR)
D
HER2.CD28ζ
% Cytotoxicity
Time (hours)
DAOY 2.0
DAOY
****
E
HER2.41BBζ
F
2 minutes
5 minutes
20 minutes
40 minutes
HER2.CD28z
HER2.41BBz
% T Cells Bound
rForce (pN)
G
CD28ζ - CD4
CD28ζ - CD8
41BBζ - CD4
41BBζ - CD8
% T cells bound
H
% T cells bound (500 pN)
***
**
I
rForce (pN)

<u>Supplementary Figure 4</u>: Analysis of protein content and mechanical properties of CARIS. **A.** Confocal microscopy of HER2-CAR T cell conjugates with HER2 amplified BT474. CARs (eGFP-green), actin (phalloidin-red), Linker for activated T cells - LAT (lipid raft surrogate-blue), Cholera toxin B subunit - CTB (binds ganglioside GM1-grey). **B.** Florescence intensity distribution of (**A**) on the CAR T cell membrane, showing colocalization at the CARIS (right). **C.** WB for membrane fractions from HER2-CAR T cells at baseline (0 min) and in conjugation with HER2 amplified BT474 at early- (15 minutes) and late- (60 minutes) CARIS. Probes are for CAR (CD3ζ), phosphorylated CARs (pY142 CD3 ζ), LAT (lipid raft marker), transferrin receptor (CD71, non-lipid raft marker). Curves represent the cholesterol concentration in each fraction. **D-E.** In xCELLigence electrical impedance cytotoxicity assays, HER2- CAR$^{CD28\zeta}$ (**D**) and CAR$^{4\text{-}1BB\zeta}$ (**E**) were added to Daoy and Daoy 2.0 (HER2 force expressed), 20 hours after tumor incubation at an E:T seeding ratio of 1:5. Statistical comparisons were done on areas under the curve (AUC). **F.** avidity curves for HER2-CAR$^{CD28\zeta}$ and HER2-CAR$^{4\text{-}1BB\zeta}$ CAR T cells after 2, 5, 20 and 40 minutes of incubation with LN229. **G.** Avidity curves for CD4 and CD8 HER2-CAR T cells after 5 minutes incubation with LN229. **H.** Percentage of CD4 and CD8 HER2-CAR T cells bound to LN229 monolayer after applying 500 pN force, after 5 minutes of incubation. **I.** Detachment forces for individual cells. Conditions were compared using one-way ANOVA with Holm-Sidak correction for multiple comparisons (D-E, and H-I). Representative of 2 donors (D-E), 3 donors (C), or 3-5 avidity runs (F-I).

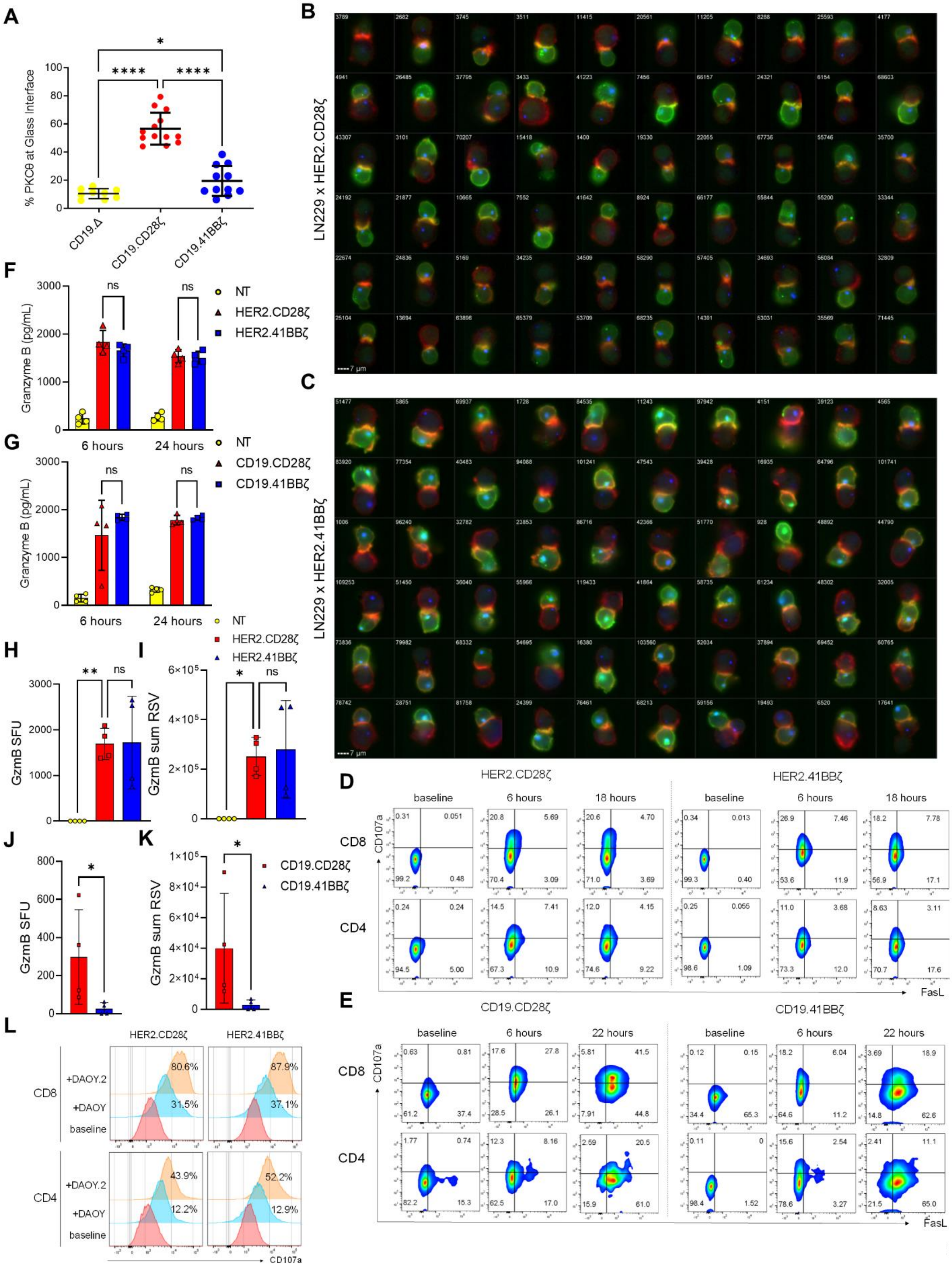

<u>Supplementary Figure 5</u>: Imaging flow cytometry of CAR T cell target interactions. A. Percentages of PKCθ (Intensity at synapse/total intensity) recruited to artificial synapses formed between CD19-CAR T cells and glass surfaces coated with CD19 recombinant protein (CD19 Fc) and anti-CD18 (IB4) antibody, after 10 minutes of incubation. Each dot represents an imaged cell. **B-C.** Representative of ImageStream for HER2-CAR$^{CD28\zeta}$ (**B**) and HER2-CAR$^{4\text{-}1BB\zeta}$ (**C**) T cells conjugated with LN229 (30 minutes). CARs (eGFP-green), actin (phalloidin-red) MTOC (pericentrin-blue) staining. Representative of 3 donors. **D-E.** Flow-cytometry for expression of the degranulation marker LAMP-1 (CD107a) and Fas ligand (FasL) on HER2- (**D**) and CD19- (**E**) CAR T cells at baseline and after coculture with LN229 for 6 or 18 hours and Daoy.CD19 for 6 and 22 hours, respectively. Representative of 3 donors. **F-G.** The supernatant of HER2-CAR T cells cocultured with LN299 (F) and of CD19-CAR T cells cocultured with Daoy.CD19 (G) were tested for granzyme B using ELISA. **H-I.** The number of granzyme B spot forming units (SPF) (H) (of total 5000 CAR T cells per well, E:T = 1:20) and the sum of relative spot volume (RSV) (I) from HER2-CAR T cells cocultured with LN229 were detected using ELISPOT. **J-K.** The number of SFU (**J**) and RSV (**K**) from 10,000 CD19-CAR T cells tested without tumor. Note: CD19-CAR T cells cocultures with DaoyCD19 produced SFU higher than the test's dynamic range. **L.** Flow-cytometry for expression of the degranulation marker LAMP-1 (CD107a) on HER2-CAR T cells at baseline and after exposure to Daoy or Daoy.2 for 5 hours. Representative of 3 donors (B-E), data from 2 donors pooled (F-K), or representative of 2 experiments (L). Conditions were compared using one-way ANOVA with Holm-Sidak correction for multiple comparisons (A, F- I), or Mann Whitney test (J-K).

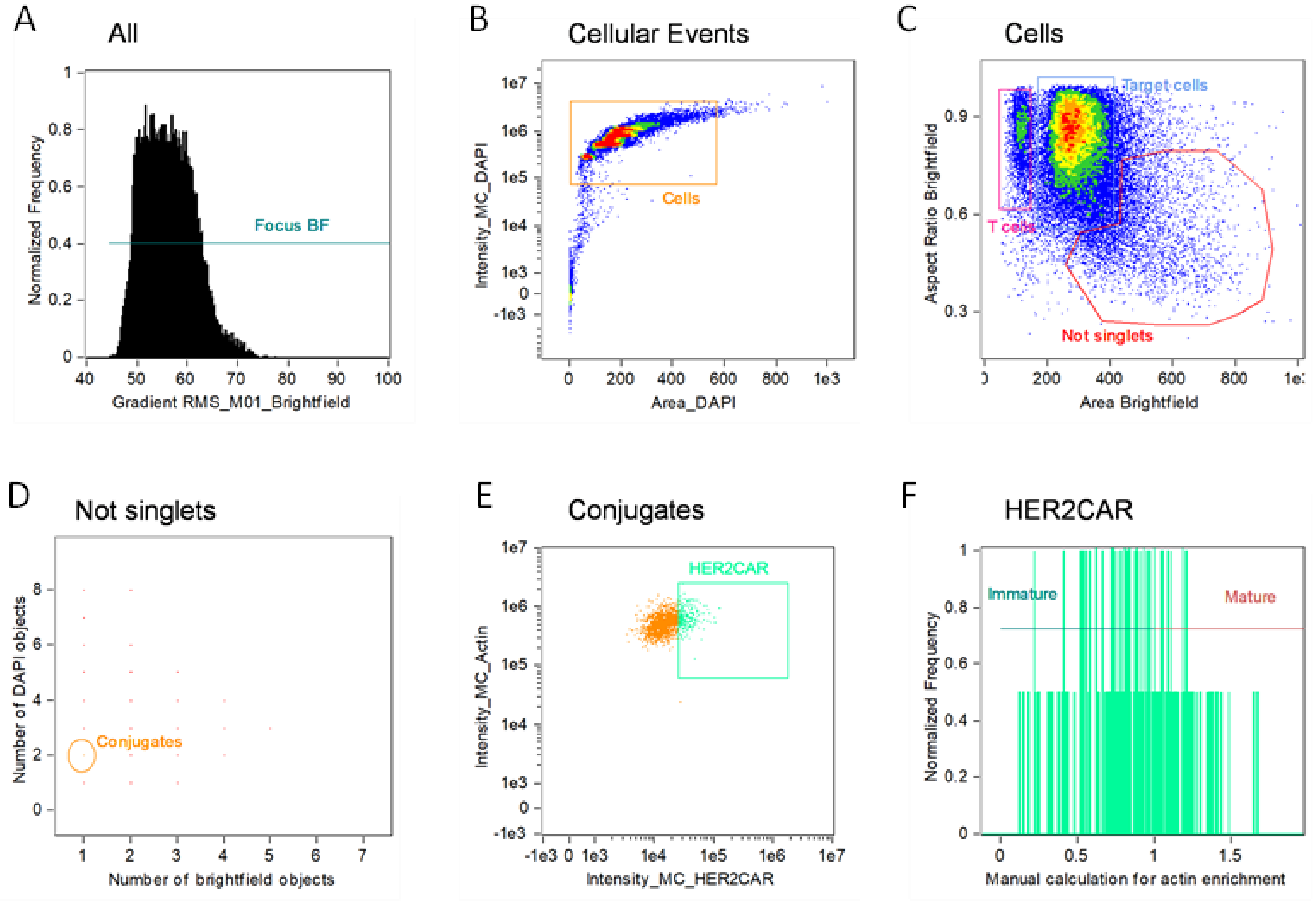

<u>Supplementary Figure 6</u>: ImageSTREAM event capture and gating strategy for CAR+ T cell and GBM conjugates: A representative ImageSTREAM flow cytometer gating strategy image taken from a co-culture of LN229 and HER2.4-1BB.ζ after 20 minutes. **A.** In focus events based on root mean square (RMS) were selected. **B.** Area and intensity of DAPI staining selected to remove non-cellular events and cell debris. **C.** Separate populations based on area to aspect ratio for singular T cells, singular target cells, and non-singular cell events. **D.** Non-singlet conjugation confirmed by DAPI object count where events have two separate nuclei labeled but appear as one item in brightfield. **E.** Conjugates with eGFP-tagged CAR+ T cells selected compared to NT controls. **F.** Actin enrichment, determined by ratio of fluorescent intensity of actin in immune synapse area divided by ratio of whole cell. “Mature synapse” defined as actin enrichment at immune synapse is over 1, “immature synapse” as less than 1.

Supplementary Video 1: Time-lapse live cell imaging for CD19-CAR$^{CD28\zeta}$ T cells in IS with Daoy.CD19.

Supplementary Video 2: Time-lapse live cell imaging for CD19-CAR$^{4\text{-}1BB\zeta}$ T cells in IS with Daoy.CD19.

Supplementary Video 3: TIMING assay for HER2-CAR T cells vs. Raji.HER2 (1 : 1)

Supplementary Video 4: TIMING assay to test for serial killing for HER2-CAR T cells vs. Raji.HER2 (1 : 2 or 3)

Supplementary Video 5: TIMING assay to test for cooperative/additive killing for HER2-CAR T cells vs. Raji.HER2 (2 : 1)

Supplementary Video 6: Time-lapse live cell imaging for MTOC / lytic granule centroid in CD19-CAR$^{CD28\zeta}$ CAR T cells in IS with Daoy.CD19.

Supplementary Table 1: LAT and CTB intensity at immune synapse. Single cell values for the intensity of Actin, CAR, CTB, and LAT at the synapse interface. CAR Design, Synapse maturation status, and donor code number are provided per event. Data attached as an xlsx file.

Supplementary Table 2: aLFA-1 intensity at immune synapse. Single cell values for the intensity of Actin, CAR, and activated LFA-1 (aLFA-1) at the synapse interface of mature synapses. CAR Design and donor code number are provided per event. Data attached as an xlsx file.

Supplementary Data 1: R Code for LAT and CTB intensity at immune synapse. The code, written using R programming, is to analyze the data in **Supplementary Table 1**, and to generate Figure 3E and 3F.

Supplementary Data 2: R code for aLFA-1 intensity at immune synapse. The code, written using R programming, is to analyze the data in **Supplementary Table 2**, and to generate Figure 4H-J.

<u>Supplementary Data 1</u>: R Code for LAT and CTB intensity at immune synapse

<u>LAT intensity at immune synapse</u>

```
library(ggplot2)
library(readr)
library(readxl)
library(dplyr)
library(tidyr)
library(gridExtra)
library(corrplot)
library(reshape2)
library(RColorBrewer)
library(ggpubr)
library(nortest)
library(scales)
library(openxlsx)

read_and_label <- function(file_1me, design, maturation, donor) {
  data <- read_excel(file_1me) %>%
    mutate(Design = design, Maturation = maturation, Donor = donor)
  return(data)
}

3_41BB_immature <- read_and_label("3_HER241BBz_LR experiment_immature.xlsx",
"HER241BBz", "Immature", "3")
3_41BB_mature <- read_and_label("3_HER241BBz_LR experiment_mature.xlsx",
"HER241BBz", "Mature", "3")
3_CD28_immature <- read_and_label("3_HER2CD28z_LR experiment_immature.xlsx",
"HER2CD28z", "Immature", "3")
```

3_CD28_mature <- read_and_label("3_HER2CD28z_LR experiment_mature.xlsx", "HER2CD28z", "Mature", "3")

1_41BB_immature <- read_and_label("1_HER241BBz_LR experiment_immature.xlsx", "HER241BBz", "Immature", "1")

1_41BB_mature <- read_and_label("1_HER241BBz_LR experiment_mature.xlsx", "HER241BBz", "Mature", "1")

1_CD28_immature <- read_and_label("1_HER2CD28z_LR experiment_immature.xlsx", "HER2CD28z", "Immature", "1")

1_CD28_mature <- read_and_label("1_HER2CD28z_LR experiment_mature.xlsx", "HER2CD28z", "Mature", "1")

2_41BB_immature <- read_and_label("2_HER241BBz_LR experiment_immature.xlsx", "HER241BBz", "Immature", "2")

2_41BB_mature <- read_and_label("2_HER241BBz_LR experiment_mature.xlsx", "HER241BBz", "Mature", "2")

2_CD28_immature <- read_and_label("2_HER2CD28z_LR experiment_immature.xlsx", "HER2CD28z", "Immature", "2")

2_CD28_mature <- read_and_label("2_HER2CD28z_LR experiment_mature.xlsx", "HER2CD28z", "Mature", "2")

2_NT_immature <- read_and_label("2_ NT_LR experiment_immature.xlsx", "NonTransduced", "Immature", "2")

2_NT_mature <- read_and_label("2_ NT_LR experiment_mature.xlsx", "NonTransduced", "Mature", "2")

1_NT_immature <- read_and_label("1_NT_LR experiment_immature.xlsx", "NonTransduced", "Immature", "1")

1_NT_mature <- read_and_label("1_NT_LR experiment_mature.xlsx", "NonTransduced", "Mature", "1")

3_NT_immature <- read_and_label("3_NT_LR experiment_immature.xlsx", "NonTransduced", "Immature", "3")

3_NT_mature <- read_and_label("3_NT_LR experiment_mature.xlsx", "NonTransduced", "Mature", "3")

```
all_data <- bind_rows(
  3_41BB_immature, 3_41BB_mature,
  3_CD28_immature, 3_CD28_mature,
  1_41BB_immature, 1_41BB_mature,
  1_CD28_immature, 1_CD28_mature,
  2_41BB_immature, 2_41BB_mature,
  2_CD28_immature, 2_CD28_mature,
  2_NT_immature, 2_NT_mature,
  1_NT_immature, 1_NT_mature,
  3_NT_immature, 3_NT_mature
)

remove_outliers_within_group <- function(data, column_1me) {
  data %>%
    group_by(Design, Maturation) %>%
    mutate(
      Q1 = quantile(.data[[column_1me]], 0.25, 1.rm = TRUE),
      Q3 = quantile(.data[[column_1me]], 0.75, 1.rm = TRUE)
    ) %>%
    ungroup() %>%
    mutate(
      IQR = Q3 - Q1,
      lower = Q1 - 1.5 * IQR,
      upper = Q3 + 1.5 * IQR
    ) %>%
    filter(.data[[column_1me]] >= lower & .data[[column_1me]] <= upper) %>%
    select(-c(Q1, Q3, IQR, lower, upper))
```

```
}

all_data_filtered <- remove_outliers_within_group(all_data, 'Intensity_Synapse interface_LAT')

all_data_filtered$Group <- factor(interaction(all_data_filtered$Design,
all_data_filtered$Maturation),
                                  levels = c("NonTransduced.Immature", "NonTransduced.Mature",
                                             "HER2CD28z.Immature", "HER2CD28z.Mature",
                                             "HER241BBz.Immature", "HER241BBz.Mature"),
                                  labels = c("Non-Transduced Immature", "Non-Transduced Mature",
                                             "HER2.CD28ζ Immature", "HER2.CD28ζ Mature",
                                             "HER2.41BBζ Immature", "HER2.41BBζ Mature"))

violin <- ggplot(all_data_filtered, aes(x = Group, y = `Intensity_Synapse interface_LAT`, fill =
Group)) +
  geom_violin(trim = FALSE) +
  geom_boxplot(width = 0.1, fill = "white", outlier.shape = 1) +
  scale_fill_manual(values = c("gray", "darkgray", "#FFCCCC", "red", "#CCCCFF", "blue")) +
  labs(title = "", x = "Group (Design x Maturation)", y = "LAT Intensity at CARIS", fill = "Group")
+
  theme_minimal() +
  theme(text = element_text(size = 18, family = "Arial"),
        axis.text.x = element_text(size = 18, family = "Arial"),
        axis.text.y = element_text(size = 35, family = "Arial"),
        axis.title.y = element_text(size = 50, margin = margin(t = 0, r = 20, b = 1, l = 3)),
        panel.grid.major = element_blank(),
        panel.grid.minor = element_blank(),
        axis.line = element_line(color = "black"),
        legend.position = "none")
```

```
print(violin)

ggsave("LAT_Intensity_with_NonTransduced.png", plot = violin, width = 9, height = 9, units = "in", dpi = 1000)


mw_test_HER241BBz <- wilcox.test(`Intensity_Synapse interface_LAT` ~ Maturation, data = filter(all_data_filtered, Design == "HER241BBz"))

mw_test_HER2CD28z <- wilcox.test(`Intensity_Synapse interface_LAT` ~ Maturation, data = filter(all_data_filtered, Design == "HER2CD28z"))

mw_test_NonTransduced <- wilcox.test(`Intensity_Synapse interface_LAT` ~ Maturation, data = filter(all_data_filtered, Design == "NonTransduced"))


print("Mann-Whitney U Test for HER241BBζ")

print(mw_test_HER241BBz)

print("Mann-Whitney U Test for HER2.CD28ζ")

print(mw_test_HER2CD28z)

print("Mann-Whitney U Test for Non-Transduced")

print(mw_test_NonTransduced)




# Q-Q Plot

ggplot(all_data_filtered, aes(sample=`Intensity_Synapse interface_LAT`)) +

  stat_qq() +

  stat_qq_line() +

  facet_grid(Design ~ Maturation)

ggplot(all_data_filtered, aes(x=`Intensity_Synapse interface_LAT`)) +

  geom_histogram(aes(y=..density..), bins=30, color="black", fill="white") +

  geom_density(alpha=.2, fill="#FF6666") +

  facet_grid(Design ~ Maturation)
```

```
ad_test_results <- all_data_filtered %>%
  group_by(Design, Maturation) %>%
  summarise(ad_p_value = ad.test(`Intensity_Synapse interface_LAT`)$p.value)
print(ad_test_results)



mw_test_HER241BBz <- wilcox.test(`Intensity_Synapse interface_LAT` ~ Maturation,
                                 data = filter(all_data_filtered, Design == "HER241BBz"))

mw_test_HER2CD28z <- wilcox.test(`Intensity_Synapse interface_LAT` ~ Maturation,
                                 data = filter(all_data_filtered, Design == "HER2CD28z"))

print(mw_test_HER241BBz)
print(mw_test_HER2CD28z)




# Collect the p-values from your tests
p_values <- c(mw_test_HER241BBz$p.value, mw_test_HER2CD28z$p.value)

# Apply Bonferroni correction
p_values_bonferroni <- p.adjust(p_values, method = "bonferroni")

# Apply Benjamini-Hochberg correction
p_values_bh <- p.adjust(p_values, method = "BH")

# Apply Holm correction
p_values_holm <- p.adjust(p_values, method = "holm")
```

```
# Print adjusted p-values
print(data.frame(
  Origi1l_p_values = p_values,
  Bonferroni = p_values_bonferroni,
  BH = p_values_bh,
  Holm = p_values_holm
))
```

CTB intensity at immune synapse

```
library(ggplot2)
library(readr)
library(readxl)
library(dplyr)
library(tidyr)
library(gridExtra)
library(corrplot)
library(reshape2)
library(RColorBrewer)
library(ggpubr)
library(nortest)
library(scales)
library(openxlsx)

read_and_label <- function(file_1me, design, maturation, donor) {
  data <- read_excel(file_1me) %>%
    mutate(Design = design, Maturation = maturation, Donor = donor)
```

```
  return(data)
}

3_41BB_immature <- read_and_label("3_HER241BBz_LR experiment_immature.xlsx", "HER241BBz", "Immature", "3")
3_41BB_mature <- read_and_label("3_HER241BBz_LR experiment_mature.xlsx", "HER241BBz", "Mature", "3")
3_CD28_immature <- read_and_label("3_HER2CD28z_LR experiment_immature.xlsx", "HER2CD28z", "Immature", "3")
3_CD28_mature <- read_and_label("3_HER2CD28z_LR experiment_mature.xlsx", "HER2CD28z", "Mature", "3")

1_41BB_immature <- read_and_label("1_HER241BBz_LR experiment_immature.xlsx", "HER241BBz", "Immature", "1")
1_41BB_mature <- read_and_label("1_HER241BBz_LR experiment_mature.xlsx", "HER241BBz", "Mature", "1")
1_CD28_immature <- read_and_label("1_HER2CD28z_LR experiment_immature.xlsx", "HER2CD28z", "Immature", "1")
1_CD28_mature <- read_and_label("1_HER2CD28z_LR experiment_mature.xlsx", "HER2CD28z", "Mature", "1")

2_41BB_immature <- read_and_label("2_HER241BBz_LR experiment_immature.xlsx", "HER241BBz", "Immature", "2")
2_41BB_mature <- read_and_label("2_HER241BBz_LR experiment_mature.xlsx", "HER241BBz", "Mature", "2")
2_CD28_immature <- read_and_label("2_HER2CD28z_LR experiment_immature.xlsx", "HER2CD28z", "Immature", "2")
2_CD28_mature <- read_and_label("2_HER2CD28z_LR experiment_mature.xlsx", "HER2CD28z", "Mature", "2")

2_NT_immature <- read_and_label("2_ NT_LR experiment_immature.xlsx", "NonTransduced", "Immature", "2")
```

```
2_NT_mature <- read_and_label("2_ NT_LR experiment_mature.xlsx", "NonTransduced",
"Mature", "2")

1_NT_immature <- read_and_label("1_NT_LR experiment_immature.xlsx", "NonTransduced",
"Immature", "1")

1_NT_mature <- read_and_label("1_NT_LR experiment_mature.xlsx", "NonTransduced",
"Mature", "1")

3_NT_immature <- read_and_label("3_NT_LR experiment_immature.xlsx", "NonTransduced",
"Immature", "3")

3_NT_mature <- read_and_label("3_NT_LR experiment_mature.xlsx", "NonTransduced",
"Mature", "3")


all_data <- bind_rows(
  3_41BB_immature, 3_41BB_mature,
  3_CD28_immature, 3_CD28_mature,
  1_41BB_immature, 1_41BB_mature,
  1_CD28_immature, 1_CD28_mature,
  2_41BB_immature, 2_41BB_mature,
  2_CD28_immature, 2_CD28_mature,
  2_NT_immature, 2_NT_mature,
  1_NT_immature, 1_NT_mature,
  3_NT_immature, 3_NT_mature
)


write.xlsx(all_data, "all_data_with_controls.xlsx", row1mes = FALSE)


CTB_outliers_included <- ggplot(all_data, aes(x = interaction(Design, Maturation), y =
`Intensity_Synapse interface_CTB`, fill = Maturation)) +
  geom_violin(trim = FALSE) +
  geom_boxplot(width = 0.1, fill = "white", outlier.shape = 1) +
  labs(title = " ", x = "Group (Design x Maturation)", y = "CTB Intensity", fill = "Maturation State")
+
```

```
  theme_minimal() +
  theme(axis.text.x = element_text(angle = 45, hjust = 1),
        legend.title = element_text(size = 12),
        legend.text = element_text(size = 10),
        axis.line = element_line(color = "black"))

print(CTB_outliers_included)
ggsave("CTB_Violen_outliers_included_with_controls.png", plot = CTB_outliers_included,
width = 9, height = 9, units = "in", dpi = 300)


remove_outliers_within_group <- function(data, column_1me) {
  data %>%
    group_by(Design, Maturation) %>%
    mutate(
      Q1 = quantile(.data[[column_1me]], 0.25, 1.rm = TRUE),
      Q3 = quantile(.data[[column_1me]], 0.75, 1.rm = TRUE)
    ) %>%
    ungroup() %>%
    mutate(
      IQR = Q3 - Q1,
      lower = Q1 - 1.5 * IQR,
      upper = Q3 + 1.5 * IQR
    ) %>%
    filter(.data[[column_1me]] >= lower & .data[[column_1me]] <= upper) %>%
    select(-c(Q1, Q3, IQR, lower, upper))
}


all_data_CTB_filtered <- remove_outliers_within_group(all_data, 'Intensity_Synapse
interface_CTB')
```

```
all_data_CTB_filtered$Group <- factor(interaction(all_data_CTB_filtered$Design,
all_data_CTB_filtered$Maturation),
                                    levels = c("NonTransduced.Immature", "NonTransduced.Mature",
                                               "HER2CD28z.Immature", "HER2CD28z.Mature",
                                               "HER241BBz.Immature", "HER241BBz.Mature"),
                                    labels = c("Non-Transduced Immature", "Non-Transduced Mature",
                                               "HER2.CD28ζ Immature", "HER2.CD28ζ Mature",
                                               "HER2.41BBζ Immature", "HER2.41BBζ Mature"))


CTB_Violen_filtered <- ggplot(all_data_CTB_filtered, aes(x = Group, y = `Intensity_Synapse
interface_CTB`, fill = Group)) +
  geom_violin(trim = FALSE) +
  geom_boxplot(width = 0.1, fill = "white", outlier.shape = 1) +
  scale_fill_manual(values = c("gray", "darkgray", "#FFCCCC", "red", "#CCCCFF", "blue")) +  #
Custom colors
  scale_y_continuous(labels = scientific) +
  labs(title = "", x = "Group (Design x Maturation)", y = "CTB Intensity at CARIS", fill = "Group")
+
  theme_minimal() +
  theme(text = element_text(size = 18, family = "Arial"),
        axis.text.x = element_text(size = 18, family = "Arial"),
        axis.text.y = element_text(size = 35, family = "Arial"),
        axis.title.y = element_text(size = 50, margin = margin(t = 0, r = 20, b = 1, l = 3)),
        panel.grid.major = element_blank(),
        panel.grid.minor = element_blank(),
        legend.position = "none",
        axis.line = element_line(color = "black"))


print(CTB_Violen_filtered)
```

```r
ggsave("CTB_Violen_outliers_removed_with_controls_ordered.png", plot =
CTB_Violen_filtered, width = 9, height = 9, units = "in", dpi = 1000)

# Perform the test for HER241BBz
mw_test_HER241BBz <- wilcox.test(`Intensity_Synapse interface_CTB` ~ Maturation,
                                 data = filter(all_data_CTB_filtered, Design == "HER241BBz"))
print("Mann-Whitney U Test for HER241BBζ")
print(mw_test_HER241BBz)

# Perform the test for HER2CD28z
mw_test_HER2CD28z <- wilcox.test(`Intensity_Synapse interface_CTB` ~ Maturation,
                                 data = filter(all_data_CTB_filtered, Design == "HER2CD28z"))
print("Mann-Whitney U Test for HER2.CD28ζ")
print(mw_test_HER2CD28z)

# Perform the test for Non-Transduced control
mw_test_NonTransduced <- wilcox.test(`Intensity_Synapse interface_CTB` ~ Maturation,
                                     data = filter(all_data_CTB_filtered, Design == "NonTransduced"))
print("Mann-Whitney U Test for Non-Transduced")
print(mw_test_NonTransduced)
```

<u>Supplementary data 2</u>: R code for aLFA-1 intensity at immune Synapse

Figure 4 codes:

```
library(ggplot2)

library(readr)

library(readxl)

library(dplyr)

library(tidyr)

library(gridExtra)

library(corrplot)

library(reshape2)

library(RColorBrewer)

library(ggpubr)

library(scales)

read_and_label <- function(file_path, design, donor) {

  data <- read_excel(file_path) %>%

    mutate(Design = design, Donor = donor)

  return(data)

}



3_41BB <- read_and_label("3_HER241BBz_LFA-1 experiment.xlsx", "HER2.41BBz", "3")

3_CD28 <- read_and_label("3_HER2CD28z_LFA-1 experiment.xlsx", "HER2CD28z", "3")

1_41BB <- read_and_label("1_HER241BBz_LFA-1 experiment.xlsx", "HER2.41BBz", "1")

1_CD28 <- read_and_label("1_HER2CD28z_LFA-1 experiment.xlsx", "HER2CD28z", "1")

2_41BB <- read_and_label("2_HER241BBz_LFA-1 experiment.xlsx", "HER2.41BBz", "2")

2_CD28 <- read_and_label("2_HER2CD28z_LFA-1 experiment.xlsx", "HER2CD28z", "2")

all_data <- bind_rows(3_41BB, 3_CD28, 1_41BB, 1_CD28, 2_41BB, 2_CD28)
```

```
if (!require(openxlsx)) {
  install.packages("openxlsx")
  library(openxlsx)
}
write.xlsx(all_data, "all_data_figure4.xlsx", row1mes = FALSE)


#combining donors
combined_data <- all_data %>%
  select(Design, `Intensity_aLFA-1 in IS`)


# Remove outliers based on the IQR method
combined_data_outliers_removed <- combined_data %>%
  group_by(Design) %>%
  mutate(Q1 = quantile(`Intensity_aLFA-1 in IS`, 0.25),
         Q3 = quantile(`Intensity_aLFA-1 in IS`, 0.75),
         IQR = Q3 - Q1,
         Lower_Bound = Q1 - 1.5 * IQR,
         Upper_Bound = Q3 + 1.5 * IQR) %>%
  filter(`Intensity_aLFA-1 in IS` >= Lower_Bound & `Intensity_aLFA-1 in IS` <= Upper_Bound)
%>%
  ungroup()
# Violin plot with outliers
p1 <- ggplot(combined_data, aes(x = factor(Design, levels = c("HER2CD28z", "HER2.41BBz"),
labels = c("HER2.CD28ζ", "HER2.41BBζ")),
                               y = `Intensity_aLFA-1 in IS`,
                               fill = factor(Design, levels = c("HER2CD28z", "HER2.41BBz"), labels =
c("HER2.CD28ζ", "HER2.41BBζ")))) +
  geom_violin(trim = FALSE) +
```

```
  geom_boxplot(width = 0.1, fill = "white", alpha = 0.5) +
  labs(title = "Comparison of aLFA-1 Intensity between HER2.CD28ζ and HER2.41BBζ (with
Outliers)",
       x = "CAR Design", y = "Intensity of aLFA-1") +
  theme_classic() +
  theme(text = element_text(size = 12),
        axis.text.x = element_text(size = 14),
        axis.text.y = element_text(size = 14),
        legend.position = "none") +
  scale_fill_manual(values = c("HER2.CD28ζ" = "red", "HER2.41BBζ" = "blue")) +
  scale_y_continuous(labels = scales::scientific_format())

print(p1)



p2 <- ggplot(combined_data_outliers_removed,
             aes(x = factor(Design, levels = c("HER2CD28z", "HER2.41BBz"),
                            labels = c("HER2.CD28ζ", "HER2.41BBζ")),
                 y = `Intensity_aLFA-1 in IS`,
                 fill = factor(Design, levels = c("HER2CD28z", "HER2.41BBz"),
                               labels = c("HER2.CD28ζ", "HER2.41BBζ")))) +
  geom_violin(trim = FALSE) +
  geom_boxplot(width = 0.1, fill = "white", alpha = 0.5) +
  labs(title = "",
       x = "CAR Design", y = "aLFA-1 Intensity at CARIS") +
  theme_classic() +
  theme(text = element_text(size = 18, family = "Arial"),
        axis.text.x = element_text(size = 30, family = "Arial"),
        axis.text.y = element_text(size = 35, family = "Arial"),
```

```
        axis.title.y = element_text(size = 50, margin = margin(t = 0, r = 10, b = 0, l = 0)),
        legend.position = "none") +
  scale_fill_manual(values = c("HER2.CD28ζ" = "red", "HER2.41BBζ" = "blue")) +
  scale_y_continuous(labels = scales::scientific_format(),
                     limits = c(1, 8e+04),
                     breaks = seq(0, 8e+04, by = 2e+04)) +
  annotate("text", x = 1.5, y = 7.6e+04, label = "p < 2.2e-16",
           size = 10, fontface = "bold", hjust = 0.8, family = "Arial")

print(p2)

ggsave("outliers_removed_CAR_comparisons_alFA_CARIS_plot_v2.png", plot = p2, width = 9, height = 9, units = "in", dpi = 300)



normality_tests_without_outliers <- combined_data_outliers_removed %>%
  group_by(Design) %>%
  summarise(normality_p_value = shapiro.test(`Intensity_aLFA-1 in IS`)$p.value,
            data_size = n())
print(normality_tests_without_outliers)
normality_tests_with_outliers <- combined_data %>%
  group_by(Design) %>%
  summarise(normality_p_value = shapiro.test(`Intensity_aLFA-1 in IS`)$p.value,
            data_size = n())
print(normality_tests_with_outliers)



MannWUtest_results <- wilcox.test(`Intensity_aLFA-1 in IS` ~ Design, data = combined_data_outliers_removed)
```

```
print(MannWUtest_results)



remove_outliers <- function(x) {
  qnts <- quantile(x, probs=c(.25, .75), 1.rm = TRUE)
  iqr <- IQR(x, 1.rm = TRUE)
  return(x[!((x < (qnts[1] - 1.5 * iqr)) | (x > (qnts[2] + 1.5 * iqr)))])
}

filtered_data <- all_data %>%
  group_by(Design) %>%
  mutate(
    `Intensity_aLFA-1 in IS` = ifelse(`Intensity_aLFA-1 in IS` %in%
remove_outliers(`Intensity_aLFA-1 in IS`), `Intensity_aLFA-1 in IS`, 1),
    `Intensity_Actin in IS` = ifelse(`Intensity_Actin in IS` %in% remove_outliers(`Intensity_Actin
in IS`), `Intensity_Actin in IS`, 1),
    `Intensity_CAR in IS` = ifelse(`Intensity_CAR in IS` %in% remove_outliers(`Intensity_CAR in
IS`), `Intensity_CAR in IS`, 1)
  ) %>%
  filter(!is.1(`Intensity_aLFA-1 in IS`), !is.1(`Intensity_Actin in IS`), !is.1(`Intensity_CAR in IS`))
%>%
  ungroup()

correlation_results <- filtered_data %>%
  group_by(Design) %>%
  summarise(
    Spearman_Correlation_Actin = cor(`Intensity_aLFA-1 in IS`, `Intensity_Actin in IS`, method
= "spearman"),
    Spearman_Correlation_CAR = cor(`Intensity_aLFA-1 in IS`, `Intensity_CAR in IS`, method =
"spearman"),
```

```
    .groups = "drop"
  )
print(correlation_results)

correlation_values <- c("HER2.41BBζ" = "0.826", "HER2.CD28ζ" = "0.809")

cor1 <- ggplot(filtered_data, aes(x = `Intensity_aLFA-1 in IS`, y = `Intensity_Actin in IS`, color = factor(Design, levels = c("HER2.41BBz", "HER2CD28z"), labels = c("HER2.41BBζ", "HER2.CD28ζ")))) +
  geom_point(alpha = 0.4) +
  geom_smooth(method = "loess", aes(group = Design)) +
  labs(title = "",
       x = "aLFA-1 Intensity at CARIS", y = "Actin Intensity at CARIS",
       color = "Design") +
  theme_minimal() +
  theme(text = element_text(family = "Arial", size = 20),
        plot.title = element_text(family = "Arial", size = 20, face = "bold"),
        axis.title = element_text(family = "Arial", size = 20),
        axis.text = element_text(family = "Arial", size = 20),
        legend.position = c(0.8, 0.2),
        legend.background = element_blank(),
        panel.grid.major = element_blank(),
        panel.grid.minor = element_blank(),
        legend.key = element_blank(),
        legend.title = element_text(family = "Arial", size = 19.5),
        legend.text = element_text(family = "Arial", size = 19.5),
        legend.direction = "vertical",
        axis.line = element_line(color = "black")) +
  scale_color_manual(values = c("HER2.41BBζ" = "blue", "HER2.CD28ζ" = "red")) +
```

```
  scale_x_continuous(labels = scientific_format()) +
  scale_y_continuous(labels = scientific_format()) +

  annotate("text", x = 4.5e4, y = 7.2e5, label = paste("Spearman HER2.41BBζ:",
correlation_values["HER2.41BBζ"]),
           vjust = 0, hjust = 1.5, size = 5, color = "blue", family = "Arial") +
  annotate("text", x = 6e4, y = 7.2e5, label = paste("Spearman HER2.CD28ζ:",
correlation_values["HER2.CD28ζ"]),
           vjust = 0, hjust = 1.0, size = 5, color = "red", family = "Arial")

print(cor1)
ggsave("correlation_plot_Actin_adjusted_higher.png", plot = cor1, width = 9, height = 9, units =
"in", dpi = 400)

# Scatter plot for LFA intensity vs. CAR intensity

correlation_values_CAR <- c("HER2.41BBζ" = "0.569", "HER2.CD28ζ" = "0.542")
cor2 <- ggplot(filtered_data, aes(x = `Intensity_aLFA-1 in IS`, y = `Intensity_CAR in IS`, color =
factor(Design, levels = c("HER2.41BBz", "HER2CD28z"), labels = c("HER2.41BBζ",
"HER2.CD28ζ")))) +
  geom_point(alpha = 0.4) +
  geom_smooth(method = "loess", aes(group = Design)) +
  labs(title = "",
       x = "aLFA-1 Intensity at CARIS", y = "CAR Intensity at CARIS",
       color = "Design") +
  theme_minimal() +
  theme(text = element_text(family = "Arial", size = 20),
        plot.title = element_text(family = "Arial", size = 20, face = "bold"),
        axis.title = element_text(family = "Arial", size = 20),
        axis.text = element_text(family = "Arial", size = 20),
```

```
      legend.position = c(0.88, 0.9),
      legend.background = element_blank(),
      legend.key = element_blank(),
      panel.grid.major = element_blank(),
      panel.grid.minor = element_blank(),
      legend.title = element_text(family = "Arial", size = 19.5),
      legend.text = element_text(family = "Arial", size = 19.5),
      legend.direction = "vertical",
      axis.line = element_line(color = "black")) +
  scale_color_manual(values = c("HER2.41BBζ" = "blue", "HER2.CD28ζ" = "red")) +
  scale_x_continuous(labels = scientific_format()) +
  scale_y_continuous(labels = scientific_format()) +
  annotate("text", x = Inf, y = Inf, label = paste("Spearman HER2.41BBζ:", correlation_values_CAR["HER2.41BBζ"]), vjust = 1.0, hjust = 2.8, size = 5, color = "blue", family = "Arial") +
  annotate("text", x = Inf, y = Inf, label = paste("Spearman HER2.CD28ζ:", correlation_values_CAR["HER2.CD28ζ"]), vjust = 1.0, hjust = 1.5, size = 5, color = "red", family = "Arial")
print(cor2)
ggsave("correlation_plot_CAR.png", plot = cor2, width = 9, height = 9, units = "in", dpi = 300)
```
